# The Immediate Effect of Sildenafil on Right Ventricular Function in Patients with Heart Failure Measured by Cardiac Magnetic Resonance: A Randomized Control Trial

**DOI:** 10.1371/journal.pone.0119623

**Published:** 2015-03-20

**Authors:** André Maurício S. Fernandes, Agnes Carvalho Andrade, Natalia Duarte Barroso, Igor Carmo Borges, Dafne Carvalho-Andrade, Erenaldo S. Rodrigues, Libia Castro Guimarães, André Rodrigues Durães, Sirlene Mendes Borges, Roque Aras

**Affiliations:** 1 Department of Cardiology, Hospital Ana Neri, Federal University of Bahia, Bahia, Brazil; 2 Department of Radiology, Hospital Ana Neri, Federal University of Bahia, Bahia, Brazil; Freeman Hospital and Newcastle University, UNITED KINGDOM

## Abstract

**Background:**

Studies have demonstrated that phosphodiesterase 5 (PDE5) inhibition is associated with right ventricle (RV) functional improvement in patients with primary pulmonary hypertension. This study aims to demonstrate the immediate impact of Sildenafil, a PDE5 inhibitor, on RV function, measured by cardiovascular magnetic resonance (CMR), in patients with heart failure (HF).

**Methods:**

We conducted a randomized double-blind controlled trial. Inclusion criteria: diagnosis of HF functional class I-III; left ventricle ejection fraction < 35%. Patients underwent CMR evaluation and were then equally randomly assigned to either 50 mg of Sildenafil or Placebo groups. One hour following drug administration, they were submitted to a second scan examination.

**Results:**

26 patients were recruited from a tertiary reference center in Brazil and 13 were allocated to each study group. The median age was 61.5 years (50–66.5 years). Except for the increase in RV fractional area change following the administration of sildenafil (Sildenafil [before vs. after]: 34.3 [25.2–43.6]% vs. 42.9 [28.5–46.7]%, p = 0.04; Placebo [before vs. after]: 28.1 [9.2–34.8]% vs. 29.2 [22.5–38.8]%, p = 0.86), there was no statistically significant change in parameters. There was no improvement in left ventricular parameters or in the fractional area change of the pulmonary artery.

**Conclusion:**

This study demonstrated that a single dose of Sildenafil did not significantly improve RV function as measured by the CMR.

**Trial Registration:**

ClinicalTrials.gov NCT01936350

## Introduction

Heart failure (HF) is a complex syndrome, involving morphologic and functional alterations in both the right and left ventricular apparatus. Although most of the HF burden is associated with left ventricular dysfunction, right ventricular (RV) impairment is also an independent predictor of morbidity and mortality in systolic HF [1]. However, there is a scarcity of data in the literature addressing RV impairment and its treatment in patients with HF.

Among the causes of RV impairment in HF, pulmonary hypertension secondary to left sided dysfunction represents the main pathophysiological mechanism [2]. Thus, therapies targeting decreased pulmonary arterial pressure and improved left ventricular function would appear to be reasonable approaches to RV improvement in HF. In this context, phosphodiesterase 5 (PDE5) inhibitors, including Sildenafil, have been considered as the mainstay for the treatment of various forms of pulmonary hypertension [3], due to their vasodilator properties on pulmonary vasculature. In addition, PDE5 inhibitors have been seen to improve left ventricular functional and structural parameters in humans with HF, and to exhibit cardioprotective activity against left ventricular remodeling, as demonstrated by several animal models of pressure-overload HF [4–8].

As well as the indirect effects on RV, PDE5 inhibition may improve the RV function of HF patients through direct mechanisms. It has been suggested that there is an up-regulation of myocardium PDE5 expression in the RV of patients with HF [9, 10] and that this is associated with severity of RV impairment [10]. PDE5 inhibition in the setting of HF and RV dysfunction has also been associated with an acute increase in myocardial contractility in *ex vivo* human [10] and animal [9] experiments. PDE5 inhibition may therefore play a role in improvement of RV function in HF.

Previous studies have demonstrated that PDE5 inhibition is associated with RV functional and geometrical improvement in patients with primary pulmonary hypertension [11, 12]. Nevertheless, when the effects of a similar treatment for RV function were evaluated in patients with pulmonary hypertension due to systolic HF, the results were varied [13–18]. In this context, this study aims to demonstrate the immediate impact of Sildenafil on RV function, as measured by cardiovascular magnetic resonance (CMR), in patients with HF.

## Materials and Methods

### Study population

The protocol for this trial and supporting CONSORT checklist are available as supporting information; see S1 CONSORT Checklist and S1 Protocol.

Patients were recruited from a tertiary reference center (Hospital Ana Neri) in Salvador, Northeast Brazil during September 2013. Inclusion criteria were: diagnosis of HF functional class I-III (New York Heart Association); left ventricle ejection fraction <35% (measured by echocardiography); and stable pharmacologic treatment for HF, prescribed by the referring physician for at least one week.

Exclusion criteria included newly diagnosed lesions during CMR evaluation (e.g., pulmonary mass); significant claustrophobia; low blood pressure (systolic blood pressure <90 mmHg or diastolic blood pressure <60 mmHg); nitrate or nebivolol use during the previous 24 hours; and the presence of non-magnetic resonance imaging-compatible implantable devices.

All patients provided written consent to participate in the study after receiving detailed information about procedures, possible clinical benefits, and risks. The study was approved by the local research ethics committee—Comitê de Ética em Pesquisa / HAN / UFBA under protocol number 118.327-2 and is registered at Clinical Trials, number NCT01936350.

### Study protocol

**Fig 1:**
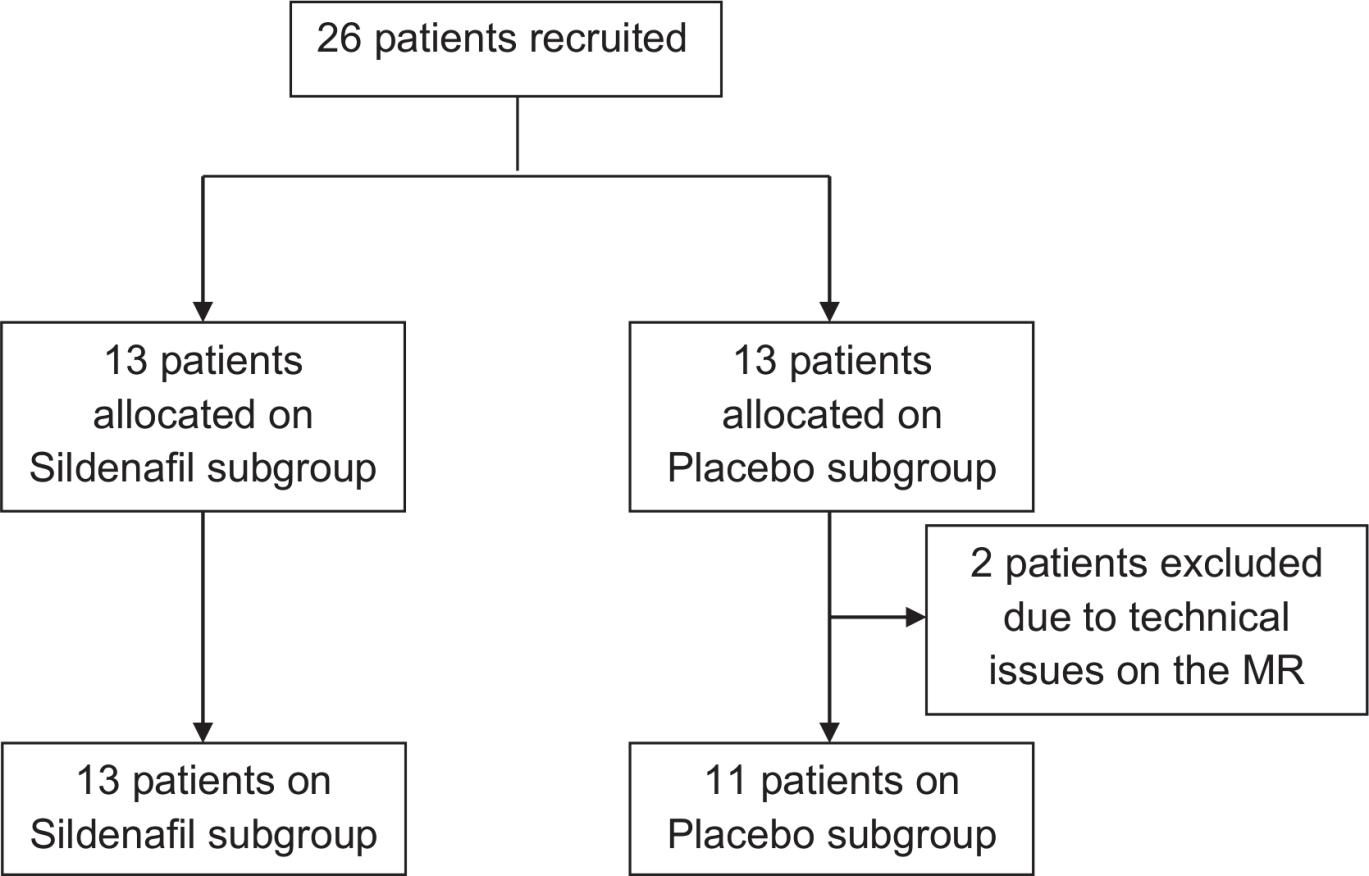
Flow chart of patients who participated in the trial. MR: magnetic resonance

Initially, patients from a HF outpatient service in a tertiary health care institution were screened for eligibility and a CMR exam was scheduled. Clinical data was then collected for all recruited patients, who underwent CMR evaluation without any intervention. Subsequently, the patients were equally randomly assigned to either the Sildenafil (50 mg of Sildenafil citrate) or Placebo groups and one hour following administration of the drug they were submitted to a second scan examination. This time lapse was selected because the median peak plasma serum concentration of sildenafil is 60 minutes. The simple randomization process was based on a computer-generated list of random numbers. Except for interventionists, nurses and technicians were kept blind to drug assignment (Fig. 1).

### CMR protocol

All subjects were scanned in the supine position. CMR was performed on an Avanto 1.5 T whole-body scanner (Siemens Medical Solutions, Germany), using an 8 channel cardiac coil. Scout images were performed to program the four-chamber, three-chamber and two-chamber, as well as short axis cine images acquisition. The cine images were all acquired using a cardiac gated multi-slice balanced steady-state free precession sequence with breath hold (20 frames per cardiac cycle at 8-mm-thick slices, FOV 300, matrix 208 Åx 80, BW 925 KHz/pixel). A stack of images, using a minimum of eight and a maximum of twelve slices, in short-axis plane with 8-mm-thick slices and a 2 mm inter-slice gap were acquired covering the entire left and right ventricles. Every effort was made to obtain adequate images with a satisfactory RV depiction.

Ventricular volume, mass, and systolic function, including RV ejection fraction (RVEF), were calculated using the cine magnetic resonance images and ARGUS 4D VF software. End-systolic frames were identified by the smallest cavity area and diastolic frames were identified by the largest cavity area. Endocardial contours were manually traced in both the systolic and diastolic frames, for at least eight slices from base to apex.

For a better depiction of RV systolic function, we measured seven septum to free wall transverse lines in the four-chamber view (for both systolic and diastolic frames). Line one was the nearest to the apex, line four was at mid-level and line seven was the baseline. These lines accurately describe regional and global RV function as described by Kind and colleagues (2010) [19]. Furthermore, the echocardiography modified tricuspid annular plane systolic excursion (TAPSE) and the RV fractional area change were measured by manually tracing the endocardial contours of the RV diastolic and systolic area and calculating percentage change. Tricuspid-annulus-apex distance change (TAAD) was calculated by manually tracing the distance between the tricuspid annulus plane and the RV apex in the four-chamber view. We also calculated the fractional TAAD (TAPSE/TAAD).

Pulmonary artery relative area change was calculated through diastolic-systolic change in the pulmonary artery area. This image was based on the RV outflow tract, where a transverse image was traced perpendicular to the artery long-axis in plane and a cine image was acquired at both the diastole and systole.

All these measurements were taken both before and after drug use.

### Statistical analyses

Based on a previous study [13], we projected an improvement in RV ejection fraction of 8% ± 4%. A 24 patient sample was thus estimated in order to provide us with 80% power to detect improvement in RVEF at a 5% two-sided level of significance.

All variables were tested for normality using the Shapiro-Wilk test. Normal continuous variables were presented as mean and standard deviation, and compared with either the unpaired Student’s t test or the paired Student’s t test, as appropriate. Variables with a non-normal distribution were described through the median and 25th-75th percentiles, and compared with the Mann-Whitney test or the paired Wilcoxon signed-rank test, as appropriate. Categorical variables were expressed as absolute values and proportions and compared with the chi-square test. Statistical analyses were performed using SPSS version 9.

A two-sided p value of 0.05 was considered the cutoff for statistical significance. The main intervention outcome was change in RVEF.

## Results

Twenty six patients were recruited and 13 were allocated to each study group. Two patients from the Placebo group were excluded, due to the impossibility of repeating their CMR exam, following technical issues with the magnetic resonance which compromised their original imaging evaluation. The Sildenafil group was therefore composed of 13 subjects, while the Placebo group contained 11 subjects (Fig. 1). Fig. 1. Flow chart of patients who participated in the trial.

Of the 24 patients included in the study, 17 (70.8%) were male and median age was 61.5 years (25^th^–75th percentile: 50–66.5 years; minimum = 33 years, maximum = 88 years). Seventeen patients (70.8%) had a systemic arterial hypertension diagnosis, 10 (41.7%) had been subject to heart catheterization, 9 (37.5%) had heart rhythm disorders, 7 (29.2%) had had myocardial infarction, and 3 (12%) had Chagas disease. Both study groups presented similar clinical characteristics (Table 1). The baseline cardiovascular parameters of the studied population are depicted in Table 2. There were no statistically significant differences in baseline cardiovascular characteristics between treatment groups.

**Table 1:** Comparison of clinical baseline characteristics between study groups.

	Sildenafil (n = 13)	Placebo (n = 11)	*P*
**Demographic**			
Gender (male)	10 (76.9)	7 (63.6)	0.66
Age (years)*	58 (49.5–64)	63 (50–68)	0.33
**Clinical**			
BMI (Kg/m^2^) *	25.5 (23.1–27.9)	23.1 (20.8–26.2)	0.20
Hypertension	10 (76.9)	7 (63.6)	0.66
Myocardial infarction	5 (38.5)	2 (18.2)	0.39
Chagas disease	2 (15.4)	1 (9.1)	1
**Medication**			
ACE Inhibitors	7 (53.8)	7 (63.6)	0.63
Beta-blockers	11 (84.6)	11 (100)	0.17
Spironolactone	10 (76.9)	8 (72.7)	0.81
Furosemide	11 (84.6)	9 (81.8)	0.86
Digoxin	9 (69.2)	8 (72.7)	0.85

BMI: body mass index. Data presented as absolute frequencies (%).

*Data presented as medians (25^th^–75^th^ percentiles).

**Table 2:** Cardiovascular baseline characteristics of the study group measured by magnetic resonance.

		Study group (24 patients)
**Right ventricle**		
RVESV (mL)		79.5 (43.5–119.3)
RVEDV (mL)		101.5 (61–161.8)
RVEF (%)		28 (17.8–33)
RV systolic area (cm^2^)		15.5 (11–21.2)
RV diastolic area (cm^2^)		21.6 (16.4–30.6)
RV fraction area change (%)		30.8 (20.3–41.2)
TAPSE (cm)		1.6 (1.2–1.9)
TAAD (cm)		7 (5.7–7.5)
Fractional TAAD (%)		0.25 (0.18–0.28)
Septum to free wall RV dimensions (mm)		
Section 1		23.65 (6.1–31.6)
Section 2		26.8 (14.3–40.6)
Section 3		30.8 (15.9–46.3)
Section 4		29.6 (16.2–36.7)
Section 5		25 (16.8–38.6)
Section 6		24.1 (16.7–34.6)
Section 7		22.9 (18.1–32)
**Pulmonary artery**		
Systolic pulmonary artery pressure (mmHg)*		48 (34.5–70)
Systolic pulmonary artery area (cm^2^)		7.8 (6.6–9.3)
Diastolic pulmonary artery area (cm^2^)		6.7 (5.7–8.2)
Pulmonary artery relative area change (%)		13 (10.6–20.8)
**Left chambers**		
LVESV (mL)		174.5 (146.5–252)
LVEDV (mL)		235 (193.3–286.8)
LVEF (%)		19.5 (14.3–24)
Left ventricular mass (g)		122 (96–136)
Septum wall thickness (cm)		0.7 (0.6–0.8)
Lateral wall thickness (cm)		0.7 (0.6–0.8)
Left atrium diameter (cm)		4.1 (3.9–5)

Data presented as median (25^th^–75^th^ percentiles). RVESV: right ventricle end-systolic volume; RVEDV: right ventricle end-diastolic volume; RVEF: right ventricle ejection fraction; RV: right ventricle; TAPSE: tricuspid annular systolic excursion; TAAD: tricuspid-annulus-apex distance change; LVESV: left ventricle end-systolic volume; LVEDV: left ventricle end-diastolic volume; LVEF: left ventricle ejection fraction.

*Systolic pulmonary artery pressure was measured by an echocardiography exam at least 6 months prior to study enrollment.

Overall, both treatment groups presented minimal changes in RV function following drug administration (Table 3 and Table 4). Except for the increase in RV fractional area change following the Sildenafil administration, neither group presented with any other statistically significant change in RV parameters, such as RVEF, fractional TAAD or septum to free wall RV dimensions.

**Table 3:** Cardiovascular effects of the interventions on treatment groups.

	Sildenafil group			Placebo group		
Variable	Before*	After*	*P* value	Before*	After*	*P* value
Right Ventricle						
RVEF (%)	29 [21–33]	26 [16–32.5]	p = 0.55	25 [16–35]	32 [11–46]	p = 0.54
RVEDV (ml)	132 [68–174]	114 [80–119]	p = 0.12	102 [44–164]	121 [64–152]	P = 0.50
RVESV (ml)	95 [50–128]	88 [52–103]	p = 0.27	77 [33–123]	91 [40–137]	P = 0.08
TAPSE						
TAAD						
Fractional TAAD (%)	0.25 [0.22–0.29]	0.30 [0.21–0.36]	p = 0.20	0.22 [0.16–0.28]	0.22 [0.19–0.32]	p = 0.72
RV fractional area change (%)	34.3 [25.2–43.6]	42.9 [28.5–46.7]	p = 0.04	28.1 [9.2–34.8]	29.2 [22.5–38.8]	p = 0.86
Pulmonary artery						
Pulmonary artery relative area change (%)	13 [9.6–21.4]	18.4 [12.9–24.7]	p = 0.21	13 [9.7–22.8]	11.9 [6.7–18.9]	p = 0.33
Left Ventricle						
LVEF (%)	20 [15–23.5]	20 [13.3–25]	p = 0.78	18 [14–25]	22 [15–25]	p = 0.62
LVEDV (mL)	242 [199–275.5]	220 [185–273]	p = 0.86	228 [154–317]	264 [197–324]	p = 0.80
LVESV(mL)	184 [152–244.5]	176 [159.5–235.5]	p = 0.81	162 [129–271]	193 [145–269]	p = 0.16

Data presented as median (25^th^–75th percentiles). RVEF: right ventricle ejection fraction; TAAD: tricuspid-annulus-apex distance change; RV: Right ventricle; LVEF: left ventricle ejection fraction; LVEDV: left ventricle end-diastolic volume; LVESV: left ventricle end-systolic volume.

* Cardiovascular parameters measured by magnetic resonance imaging at baseline (before) and one hour following the administration of Sildenafil or the placebo pill (after).

**Table 4:** Absolute right ventricle parameters changes before and after the sildenafil and placebo

Variable Mean (SD)	Sildenafil treatment group (n = 13)	Placebo treatment group (n = 11)	Difference between groups	95% confidence interval	*P* value
RVEF	−2.3 (14.7)	+3.3(13.2)	−5.6	−17.5 to 6.3	0.34
RVEDV	−18.2 (37.2)	+19.6 (27.8)	−37.8	−66.0 to −9.5	0.01
RVESV	−6.9 (34.6)	+14.5 (21.8)	−21.4	−46.4 to 3.6	0.91
TAPSE	+0.22 (0.6)	+0.14 (0.4)	0.08	−0.4 to 0.5	0.74
Fractional TAAD	+0.04 (0.1)	+0.01 (0.08)	0.03	−0.05 to 0.1	0.47
RV fractional area change	+4.9 (7.2)	+1.4 (10.7)	3.5	−4.1 to 11.1	0.35
Pulmonary artery relative area change	+1.3 (12.9)	−1.9 (5.4)	3.2	−5.2 to 11.5	0.43

RVEF: right ventricle ejection fraction; TAAD: tricuspid-annulus-apex distance change; RV: Right ventricle; LVEF: left ventricle ejection fraction; LVEDV: left ventricle end-diastolic volume; LVESV: left ventricle end-systolic volume.

None of the studied interventions significantly altered pulmonary artery relative area change (Table 3).

Left ventricle parameters did not change significantly after either of the two interventions. Functional parameters such as left ventricle ejection fraction (LVEF), left ventricle end-diastolic volume (LVEDV) and left ventricle end-systolic volume (LVESV) altered only minimally (Table 3).

There were no adverse effects related to the administration of either Sildenafil or placebo pills.

## Discussion

The study demonstrated that a single dose of 50 mg of Sildenafil citrate causes minimal, if any, change in RV function as measured by CMR in patients with HF. The main outcome of the investigation, change in RVEF, did not demonstrate any effect due to the studied intervention. This result was confirmed by the evaluation of other RV function parameters, such as fractional TAAD and septum to free wall RV dimensions. Although the increase in RV fractional area change following Sildenafil administration suggests an improvement in RV function associated with the intervention, this is a less reliable parameter for RV function and may reflect minor changes in RV function or geometrical RV changes unrelated to its function. In general, RV fractional area change underestimates MRI derived volume and is inferior to using 3D echo to estimate RV volume, since it does not evaluate right ventricle out flow.

There is an intrinsic problem in determining RV function. RV myocardial fibers have a longitudinal orientation giving them a distinctive longitudinal contraction and this complex, three dimensional shape cannot be compared to any geometrical form. This means that, due to a range of geometric assumptions, any two dimensional measurement, such as RV fractional area change and TAPSE, may be misleading [20].

Previous studies have confirmed the effect of sildenafil on right ventricular function in patients with pulmonary hypertension, especially in relation to long-term use [11,12]. Nevertheless, this effect is uncertain in patients with right systolic dysfunction due to left sided ventricle impairment. Two previous studies corroborate the negligible ability of Sildenafil to improve RV function in patients with HF. A case-control study that evaluated 32 patients with HF treated with Sildenafil (mean dose 75 ± 25 mg per day) for 3 months did not demonstrate changes in RV end-diastolic diameter or RV dysfunction grade, as measured by echocardiography following intervention [18]. Further, a retrospective study that evaluated 16 patients with HF and treated them with a mean dose of 102.5 ± 54 mg of Sildenafil per day for 6 months did not find changes in the right ventricular stroke work index, as measured by right heart catheterization [17].

Controversially, a small number of previous studies have demonstrated that Sildenafil was able to improve the RV function of patients with HF. These demonstrated that both a single dose of Sildenafil (50 mg) and continued use of this medication (60–225 mg per day) improved the functional parameters of the RV, such as RVEF (measured by right heart catheterization), TAPSE, and right ventricle end-diastolic diameter (measured by echocardiography) [13–16].

There is no clear explanation for the contradiction in findings regarding the efficacy of Sildenafil to improve the RV function of HF patients. One possibility may be related to the HF etiology in the patients enrolled in these studies. Evidence has been found of an etiology-dependent PDE5 expression in the RV myocytes of patients with HF [10] and this may influence the response to Sildenafil treatment. The regulation of PDE5 RNA expression differs in normal and hypertrophied RV. A previous study has demonstrated that in the whole heart, and at even in a cardiomyocyte, there is an increase in contractility with PDE inhibition for right ventricle hypertrophy [9] but not for those with normal ventricular function, such as the patients in this study. This suggests that the effect of sildenafil is probably minor or restricted to certain groups of patients, which may explain the absence of effect in this population, which has impaired RV function but normal RV volume. Another explanation may be related to investigational procedures and parameters analyzed. Most of the studies that demonstrate RV improvement used invasive techniques for RVEF evaluation, whereas most that did not demonstrate such an effect used non-invasive techniques, while other studies did not assess changes in RVEF. However, this study used a reliable non-invasive technique for RV function measurement and did not find any changes in RVEF associated with Sildenafil administration. These factors suggest that Sildenafil might able to improve the RV function of patients with HF, highlighting a process that is incompletely understood. As demonstrated in previous studies, this effect is probably minor and/or restricted to a certain group of patients.

The absence of improvement in left ventricular parameters and in the fractional area change of the pulmonary artery in this study probably reflect the same rationale that applies to the minimal changes in the parameter for RV function. Thus, these findings reinforce the minimal impact of a single dose of Sildenafil on cardiac function.

Certain study limitations should be addressed. Despite the accuracy of CMR in evaluating heart function, the effect of Sildenafil on systolic function may be so small as to fall within the normal variation that may result from two points of evaluation. The study used a 50mg dose of Sildenafil citrate and it is possible that a 100mg dose could have had an impact on RV function. Furthermore, this was not a prospective study and did not evaluate the impact of the long-term use of Sildenafil.

This study demonstrated that a single dose of Sildenafil did not significantly improve RV function as measured by the CMR of patients with HF. Since controversies persist regarding the effect of Sildenafil on cardiac function in the setting of HF, more studies are required to elucidate this issue, particularly in certain groups of patients with heart failure.

## Supporting Information

S1 CONSORT Checklist Fulfilled consort checklist. (DOC)

S1 Protocol Detailed project in English. (DOC)

S2 Protocol Detailed project in original language. (DOC)

S1 Sildenafil Data Bank Major study data bank. (XLSX)

S1 IRB Approval Document from local institutional review board with the study ethical approval. (PDF)
