# Supplementary material for: The Immediate Effect of Sildenafil on Right Ventricular Function in Patients with Heart Failure Measured by Cardiac Magnetic Resonance: A Randomized Control Trial
Source: PLoS One. 2015 Mar 20;10(3):e0119623. doi: 10.1371/journal.pone.0119623 (PMC4368670; doi:10.1371/journal.pone.0119623)
Supplement: S1 Protocol — (DOC) [file pone.0119623.s002.doc]

| 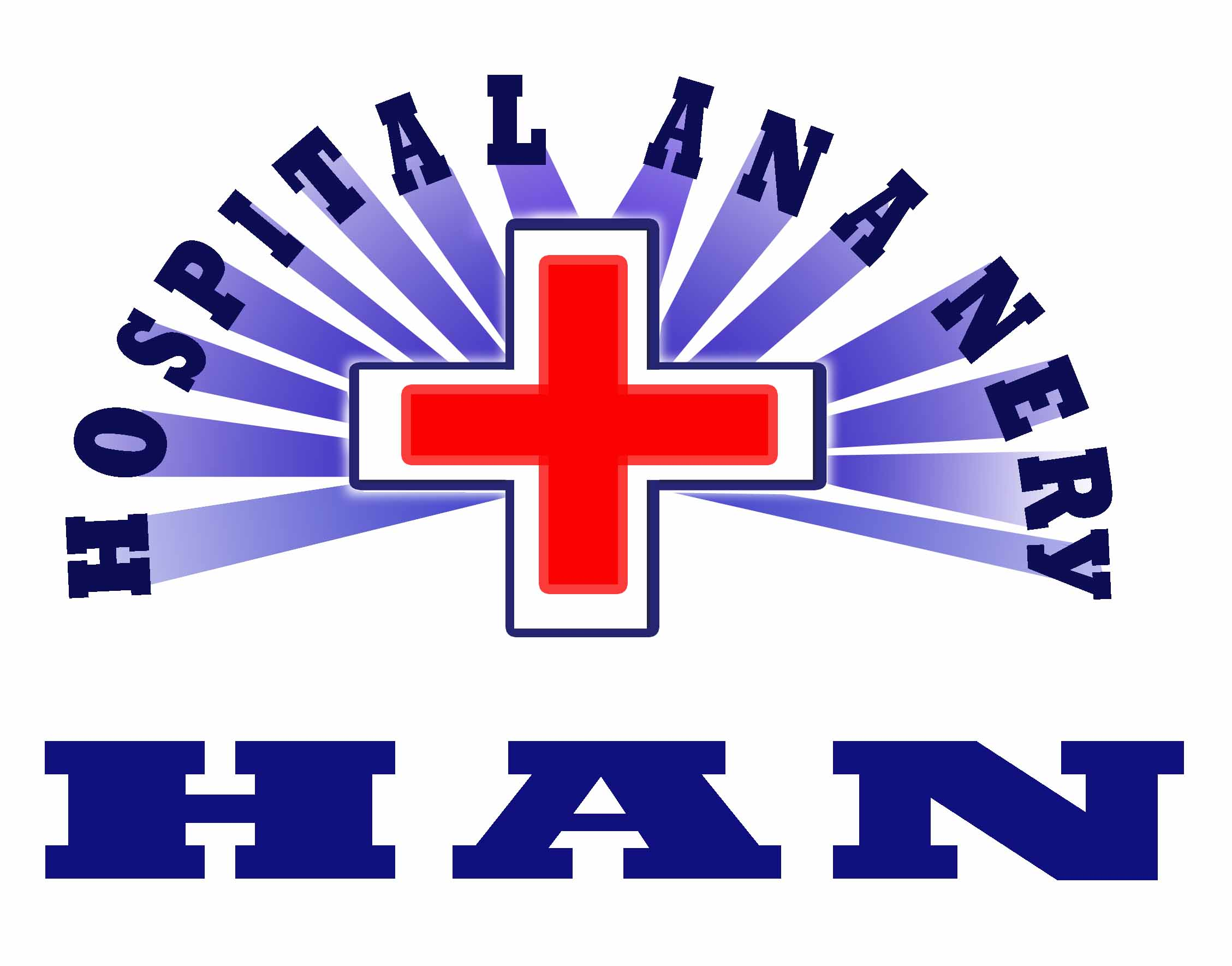 | **HOSPITAL ANA NERI** | **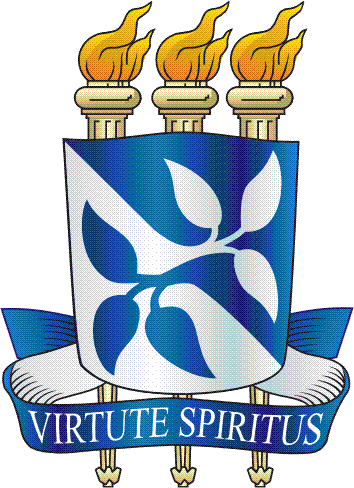** |
| --- | --- | --- |
| HAN |  | **UFBA** |

Sildenafil Impact on Ventricular Function in Patients With Heart Failure: Randomized Clinical Trial

André Maurício Souza Fernandes

Salvador - BA

2012

**Title:** Sildenafil Impact on Ventricular Function in Patients With Heart Failure: Randomized Clinical Trial

Hospital Ana Neri

Setor de Bioimagem do HAN

- **Pesquisador: ANDRÉ MAURÍCIO SOUZA FERNANDES,** Médico cardiologista. Preceptor da unidade de internação de cardiologia do HAN – UFBA [andremsf@hotmail.com](mailto:andremsf@hotmail.com)
- **Colaboladores:**

**ROQUE ARAS JÚNIOR**, Médico cardiologista. Diretor médico do HAN. Professor adjunto IV do departamento de Medicina da UFBA.

**SIRLENE MENDES BORGES**, Médica radiologista. Coordenadora do setor de bioimagem do HAN.

**ALISSON DE AQUINO FIRMINO**, Biomédico. Funcionário do setor de bioimagem do HAN.

**DAFNE CARVALHO ANDRADE**, Estudante da Faculdade de Medicina da Bahia da UFBA, cursando o quinto ano do curso em 2012.

**IGOR CARMO BORGES**, Estudante da Faculdade de Medicina da Bahia da UFBA, cursando o quinto ano do curso em 2012.

**AGNES CARVALHO ANDRADE**, Estudante da Faculdade de Medicina da Bahia da UFBA, cursando o terceiro ano do curso em 2012.

**NATÁLIA DUARTE BARROSO**, Estudante da Faculdade de Medicina da Bahia da UFBA, cursando o terceiro ano do curso em 2012.

**ÍNDICE**

I. QUESTION

II. INTRODUCTION

III. AIMS

IV. MATERIAL AND METHODS

V. ETHICAL ISSUES

VI. REFERENCES

VII IMPACT

X. ATTACHES

**I. QUESTION**

What is the impact of a single dose of sildenafil on right ventricular function on patients with cardiac failure?

**II. INTRODUCTION**

Heart failure (HF) is a complex syndrome, involving morphologic and functional alterations in both the right and left ventricular apparatus. Although most of the HF burden is associated with left ventricular dysfunction, right ventricular (RV) impairment is also an independent predictor of morbidity and mortality in systolic HF [1]. However, there is a scarcity of data in the literature addressing RV impairment and its treatment in patients with HF.

Among the causes of RV impairment in HF, pulmonary hypertension secondary to left sided dysfunction represents the main pathophysiological mechanism [2]. Thus, therapies targeting decreased pulmonary arterial pressure and improved left ventricular function would appear to be reasonable approaches to RV improvement in HF. In this context, phosphodiesterase 5 (PDE5) inhibitors, including Sildenafil, have been considered as the mainstay for the treatment of various forms of pulmonary hypertension [3], due to their vasodilator properties on pulmonary vasculature. In addition, PDE5 inhibitors have been seen to improve left ventricular functional and structural parameters in humans with HF, and to exhibit cardioprotective activity against left ventricular remodeling, as demonstrated by several animal models of pressure-overload HF [4-8].

As well as the indirect effects on RV, PDE5 inhibition may improve the RV function of HF patients through direct mechanisms. It has been suggested that there is an up-regulation of myocardium PDE5 expression in the RV of patients with HF [9, 10] and that this is associated with severity of RV impairment [10]. PDE5 inhibition in the setting of HF and RV dysfunction has also been associated with an acute increase in myocardial contractility in *ex vivo* human [10] and animal [9] experiments. PDE5 inhibition may therefore play a role in improvement of RV function in HF.

Previous studies have demonstrated that PDE5 inhibition is associated with RV functional and geometrical improvement in patients with primary pulmonary hypertension [11, 12]. Nevertheless, when the effects of a similar treatment for RV function were evaluated in patients with pulmonary hypertension due to systolic HF, the results were varied [13-18]. In this context, this study aims to demonstrate the immediate impact of Sildenafil on RV function, as measured by cardiovascular magnetic resonance (CMR), in patients with HF.

**III. AIM**

TO EVALUATE THE impact of a single dose of sildenafil on right ventricular function on patients with cardiac failure.

**IV. MATERIAL E METHODS**

**1. Local do estudo**

This study will be performed at the magnetic resonance unit at the ANA NERI HOSPITAL – SALVADOR – BAHIA - BRAZIL

**2. Métodos**

2.1 Study population

Patients were recruited from a tertiary reference center (Hospital Ana Neri) in Salvador, Northeast Brazil. Inclusion criteria were: diagnosis of HF functional class I-III (New York Heart Association); left ventricle ejection fraction <35% (measured by echocardiography); and stable pharmacologic treatment for HF, prescribed by the referring physician for at least one week.

All patients provided written consent to participate in the study after receiving detailed information about procedures, possible clinical benefits, and risks. The study was approved by the local research ethics committee under protocol number 118.327-2 and is registered at Clinical Trials, number NCT01936350.

2.1 study design

This is a double blind randomized clinical trial.

2.2 Cálculo da amostra

O tamanho da amostra calculado para cada grupo foi de 12 participantes, totalizando 24 participantes no estudo. O cálculo amostral foi realizado baseado no estudo de Lewis et al (2007), considerando a diferença a ser detectada na comparação da fração de ejeção do ventrículo direito de 8%, desvio padrão de 4%, nível de significância de 0,1%, poder do teste de 95% e teste de hipótese bicaudado

2.3 Inclusion criteria

Patients were recruited from a tertiary reference center (Hospital Ana Neri) in Salvador, Northeast Brazil. Inclusion criteria were: diagnosis of HF functional class I-III (New York Heart Association); left ventricle ejection fraction <35% (measured by echocardiography); and stable pharmacologic treatment for HF, prescribed by the referring physician for at least one week.

2.4 Exclusion criteria

Exclusion criteria included newly diagnosed lesions during CMR evaluation (e.g., pulmonary mass); significant claustrophobia; low blood pressure (systolic blood pressure <90 mmHg or diastolic blood pressure <60 mmHg); nitrate or nebivolol use during the previous 24 hours; and the presence of non-magnetic resonance imaging-compatible implantable devices.

2.5 Sequência de atendimento:

Patients will answer some question about their clinical history Os pacientes responderão a um questionário sobre dados clínicos e sociodemográficos e depois serão submetidos a RMC antes e após 1 hora de uso de 50 mg de citrato de sildenafil. As imagens da RMC serão obtidas utilizando um magneto de corpo inteiro de 1.5T Avanto Siemens.

All subjects were scanned in the supine position. CMR was performed on an Avanto 1.5 T whole-body scanner (Siemens Medical Solutions, Germany), using an 8 channel cardiac coil. Scout images were performed to program the four-chamber, three-chamber and two-chamber, as well as short axis cine images acquisition. The cine images were all acquired using a cardiac gated multi-slice balanced steady-state free precession sequence with breath hold (20 frames per cardiac cycle at 8-mm-thick slices, FOV 300, matrix 208 Åx 80, BW 925 KHz/pixel). A stack of images in short-axis plane with 8-mm-thick slices and a 2 mm inter-slice gap were acquired covering the entire left and right ventricles. Every effort was made to obtain adequate images with a satisfactory RV depiction.

Ventricular volume, mass, and systolic function, including RV ejection fraction (RVEF), were calculated using the cine magnetic resonance images and ARGUS software. End-systolic frames were identified by the smallest cavity area and diastolic frames were identified by the largest cavity area. Endocardial contours were manually traced in both the systolic and diastolic frames, at least eight slices from base to apex.

For a better depiction of RV systolic function, we measured seven septum to free wall transverse lines in the four-chamber view (for both systolic and diastolic frames). Line one was the nearest to the apex, line four was at mid-level and line seven was the baseline. These lines accurately describe regional and global RV function as described by Kind and colleagues (2010) [19]. Furthermore, the echocardiography modified tricuspid annular plane systolic excursion (TAPSE) and the RV fractional area change were measured by manually tracing the endocardial contours of the RV diastolic and systolic area and calculating percentage change. Tricuspid-annulus-apex distance change (TAAD) was calculated by manually tracing the distance between the tricuspid annulus plane and the RV apex in the four-chamber view. We also calculated the fractional TAAD (TAPSE/TAAD).

Pulmonary artery relative area change was calculated through diastolic-systolic change in the pulmonary artery area. This image was based on the RV outflow tract, where a transverse image was traced perpendicular to the artery long-axis in plane and a cine image was acquired at both the diastole and systole.

All these measurements were taken both before and after drug use.

2.4 Statistical analyses

Based on a previous study [13], we projected an improvement in RV ejection fraction of 8% ± 4%. A 24 patient sample was thus estimated in order to provide us with 80% power to detect improvement in RVEF at a 5% two-sided level of significance.

All variables were tested for normality using the Shapiro-Wilk test. Normal continuous variables were presented as mean and standard deviation, and compared with either the unpaired Student’s t test or the paired Student’s t test, as appropriate. Variables with a non-normal distribution were described through the median and 25th-75th percentiles, and compared with the Mann-Whitney test or the paired Wilcoxon signed-rank test, as appropriate. Categorical variables were expressed as absolute values and proportions and compared with the chi-square test. Statistical analyses were performed using SPSS version 9.

A two-sided p value of 0.05 was considered the cutoff for statistical significance. The main intervention outcome was change in RVEF.

Os indivíduos serão examinados em decúbito dorsal e a recepção do sinal era obtida através de uma bobina de 4 canais. Os seguintes parâmetros serão utilizados: tempo de repetição (TR) = 9ms, tempo de eco (TE) = 4ms, ângulo flip (FA) = 40 graus, espessura de corte = 8mm, número de excitações (NEX) = 2-4, no domínio da view = 380 - 420mm, matriz de 128 x128. Imagens guias em cortes axial, coronal e sagital serão usadas para planejar seqüências multiplanares com precessão livre dos prótons que correspondem a imagens dinamicas do coração (eixo curto, eixo horizontal

longo, vertical longo ). Em nenhum momento do estudo os pacientes serão submetido a radiação ionizante e não haverá uso de qualquer tipo de contraste. Após a aquisição de imagens, serão realizadas as medidas funcionais do ventrículo esquerdo (VE) e do ventrículo direito (VD) (Guideline RM e TC, 2004). Imediatamente e 1 hora após o uso de 50 mg de sildenafil, o paciente será questionado quanto à presença de efeitos adversos ao uso da droga. O médico avaliador da ressonância magnética não terá contato com o paciente e o mesmo será identificado na plataforma do exame por um número anteriormente cadastrado pela equipe de pesquisa. Estando esse, portanto, “cego” ao uso ou não da medicação. O paciente que apresentar intercorrência durante o exame, efeito colateral do uso da medicação ou claustrofobia será atendido pelo médico do setor e excluído da amostra do estudo, já que o paciente deixou de preencher o critério “cego” do estudo.

2.6 Variáveis de interesse:

Dados clínicos e sociodemográficos: idade, sexo, raça, história de sintomas, hábitos de vida, diagnósticos prévios, antecedentes familiares, tratamento medicamentoso, freqüência cardíaca, pressão arterial, peso, altura, medida de cintura abdominal, alergias medicamentosas e presença de reação adversa ao uso de citrato de sildenafil.

Resultado da ressonância magnética antes e após 50mg de sildenafil: fração de ejeção do VE e do VD, volumes sistólico e diastólico do VE e do VD, diâmetros sistólico e diastólico do VE e do VD, análise de contratilidade e torção ventricular, TAPSE (*tricusped annular plane systolic excursion*), massa ventricular esquerda e direita, volume de regurgitação tricúspide e mitral, curvatura septal, pressão no ventrículo direito, débito cardíaco, fluxo pulmonar e distensibilidade da artéria pulmonar.

2.7 Hipóteses:

Hipótese nula: uma dose oral de 50mg de citrato dsildenafil não altera a função ventricular direita de pacientes com insuficiência cardíaca.

Hipótese alternativa: uma dose oral de 50mg de citrato de sildenafil não altera a função ventricular direita de pacientes com insuficiência cardíaca.

**3. Análise estatística**

Serão avaliadas as correlações entre as variáveis clínicas, sociodemográficas e do resultado da ressonância magnética cardiovascular dos pacientes antes e após 50mg de sildenafil. Serão também descritos os dados coletados por meio de médias e desvios-padrões ou números absolutos e números relativos. A análise estatística será desempenhada com auxilio do software SSPS (versão 9.0), com a realização de testes estatísticos como: teste t de Student, Mann Whitney, teste t pareado, teste dos sinais de Wilcoxon, teste qui quadrado, teste exato de Fisher, Kruskall Wallis, one-way ANOVA, teste de correlação de Pearson e regressão linear e logística, simples ou multivariada.

**V. ASPECTOS ÉTICOS**

O trabalho será submetido ao Comitê de Ética em Pesquisa do HAN. Quando na publicação dos resultados, nenhum paciente será identificado e o HAN será citado na publicação, não inferindo qualquer tipo de maleficência ao paciente decorrente aos dados publicados. No final do estudo, será encaminhado ao Comitê de Ética em Pesquisa relatório final e cópia do trabalho que será publicado.

Possíveis efeitos adversos relacionados ao uso de sildenafil incluem cefaléia, *flushing*, hipotensão, turvação visual, tontura, epistaxe, diarréia e exantema. Normalmente, nenhum dos efeitos adversos associados ao uso da droga é grave, não representando perigo para a vida dos participantes do presente estudo. Caso o participante apresente algum sintoma associado ao uso da droga em questão, este será mantido em observação pela equipe do estudo até melhora completa ou estabilização do quadro, sendo encaminhado para o pronto atendimento da instituição para estabilização clínica, caso necessário.

**VI. REFERÊNCIAS BIBLIOGRÁFICAS**

**VII. EXEQUIBILIDADE**

O setor de bioimagem do HAN dispõe de um aparelho de ressonância magnética 1.5T recentemente adquirido, sendo o único aparelho em um hospital exclusivamente público capaz de realizar ressonância magnética cardiovascular no estado da Bahia.

A equipe de pesquisadores dispõe de recurso financeiro pessoal para aquisição das medicações usadas no estudo.

**VIII. APLICABILIDADE**

Ainda não há na literatura estudos mostrando o efeito do citrato de sildenafil na função ventricular direita medida através da RMC.

**IX. IMPACTO**

Imediato. Achados desse estudo podem ter impacto de grande importância no tratamento do pacientes com IC.

**X. APÊNDICES**

Apêndice 01

**CRONOGRAMA**

| **Atividades** | **Mai/12** | **Jun/12** | **Jul/12** | **Jul/12-Dez/12** | **Jan/13** | **Fev/13** | **Mar/13** |
| --- | --- | --- | --- | --- | --- | --- | --- |
| Revisão da literatura | x | x |  |  |  |  |  |
| Construção do projeto e submissao ao CEP | x | x | x |  |  |  |  |
| Coleta de dados |  |  | x | x |  |  |  |
| Análise de dados |  |  |  |  | x | x |  |
| Confecção do manuscrito |  |  |  |  | x | x |  |
| Revisão do manuscrito |  |  |  |  |  | x | x |
| Submissão à publicação |  |  |  |  |  | x | x |

Apêndice 02

**ORÇAMENTO**

Despesas de capital:

1. Aparelho de ressonância magnética de corpo inteiro de 1.5 T Avanto Siemens (disponível no HAN)

2. Notebook LG R480L-3100 c/ Intel® Pentium Dual Core T4300 2.1GHz 3GB 320GB DVD-RW Webcam 1.3MP LED 14" Windows 7 Basic – LG (disponível pelo pesquisador André Maurício Souza Fernandes)

Subtotal: R$ 0

Despesas correntes:

Descrição Qnt Valor Unit Valor Total

Citrato de sildenafil 12 R$ 10,00 R$ 120,00

Placebo 12 R$ 5,00 R$ 60,00

Impressão de material. 50 R$ 0,20 R$ 10,00

Fotocópia 300 R$ 0,10 R$ 30,00

Pasta de arquivos 2 R$ 20,00 R$ 40,00

Subtotal: R$ 260,00

Total: R$ 260,00

Apêndice 03

**TERMO DE CONSENTIMENTO LIVRE E ESCLARECIDO**

Eu,_____________________________________________________________________(nome),______________________(nacionalidade),________(idade), ________________ (estado civil), ___________________(profissão),_______________________________________________________________ (endereço), _______________ (RG), estou sendo convidado a participar de um estudo denominado:

**Impacto do sildenafil na função ventricular direita de pacientes com insuficiência cardíaca**

Fui informado que tenho insuficiência cardíaca, um problema na função de bombear o sangue pelo coração, e que estou sendo convidado a participar de um estudo no qual farei uso de um medicamento que contém citrato de sildenafil ou uma substância sem efeito significativo (plaebo) e também serei submetido a um exame chamado ressonância magnética cardiovascular (um tipo de fotografia especial do coração) antes e uma hora após usar esse medicamento. Este estudo tem como objetivo avaliar o efeito do citrato de sildenafil na função do meu coração.

Fui informado que possíveis efeitos colaterais causados pelo citrato de sildenafil incluem dor de cabeça, vermelhidão na face, pressão baixa, vista embaçada, tontura, sangramento no nariz, diarréia e “empolação” na pele. Informaram também que, normalmente, nenhum dos efeitos colaterais causados pelo medicamento é grave, não representando perigo para minha vida. Fui assegurado que caso eu apresente algum sintoma associado ao uso do medicamento, eu ficarei em observação pela equipe do estudo até melhora completa ou estabilização do quadro, sendo encaminhado para o pronto atendimento do hospital para melhora clínica, caso necessário. Ainda avisaram que caso eu apresente claustrofobia (medo de ficar em lugares fechados) durante o exame de ressonância magnética, o exame será interrompido. O desconforto que poderei sentir durante a entrevista é da possibilidade de compartilhar um pouco das minhas informações pessoais ou confidenciais. Contudo, não precisarei responder qualquer pergunta na entrevista se eu sentir que ela é muito pessoal ou se sentir incômodo ao falar. As informações que estão previstas para serem respondidas no questionário são sobre minha saúde, meus medicamentos e exames que fiz anteriormente.

Estou ciente que minha privacidade será respeitada, ou seja, meu nome ou qualquer outro dado ou elemento que possa, de qualquer forma, me identificar, será mantido em sigilo.

Também fui informado de que posso me recusar a participar do estudo, a qualquer momento, sem precisar justificar, não sofrendo qualquer prejuízo à assistência médica. Os pesquisadores também têm o direito de cancelar minha participação no estudo a qualquer momento.

Foi garantido que terei livre acesso a todas as informações e esclarecimentos adicionais necessários e que o pesquisador se compromete a deixar uma cópia do relatório final da pesquisa nesta instituição para disposição de todos, e fará a divulgação dos resultados obtidos através de eventos e revistas científicas nacionais e internacionais.

Fui informado que todas as despesas com a pesquisa, bem como o ressarcimento de gastos decorrentes dos riscos e complicações causados pelo uso do medicamento e realização da entrevista serão de total responsabilidade do pesquisador. Os dados obtidos serão armazenados por um período de cinco anos. Este termo de consentimento livre e esclarecido será assinado por mim em duas vias, com o compromisso do pesquisador de me proporcionar uma cópia do mesmo para meu controle.

Conforme determina a Resolução 196/96, do Conselho Nacional de Saúde, que trata de aspectos éticos da pesquisa de enfermagem envolvendo seres humanos, o presente estudo requer a participação voluntária dos sujeitos.

Afirmo que a minha participação é voluntária, o meu consentimento para participar da pesquisa foi de livre decisão, não tendo sofrido nenhuma interferência do pesquisador. Estou ciente de que não serei remunerada (o) por este ato, de que poderei solicitar o pesquisador para rever as informações que forneci na entrevista, estando livre para corrigir parte do que foi dito por mim, além de me recusar a continuar participando do estudo a qualquer momento sem causar nenhum prejuízo a minha pessoa e nem a meu futuro profissional.

**Salvador, ____ de _________________ de 2012**

___________________________________ ________________________________

Assinatura do Participante da Pesquisa Assinatura do Pesquisador

Dados do pesquisador

Nome: Dr. André Maurício Fernandes
E-mail: andremsf@hotmail.com

Telefone: 3117-1923/1903

Endereço: Rua Saldanha Marinho, s/n - Caixa d'Água  Salvador - BA, 40320-010

Apêndice 04

**FICHA CLÍNICA**

| **Identificação** |
| --- |
| 1. Data: / /  2. Número do registro [ ] [ ] [ ] [ ] [ ] [ ] Externo [ ]  3. Nome ___________________________________________________________________________  4. Data de Nascimento _______/_______/________  5. Idade _________  6. Gênero [ ] (1-feminino, 2-masculino)  7. Grupo racial [ ] (1-Branco, 2-pardo, 3-negro, 4-outro)  8. Telefone: (____)________________________/(____)_________________________ |
| **Historia de Sintomas (1-Sim; 2- Não) *últimos 3 meses** |
| 9. Dispnéia [ ]  10. Edema de MMII [ ]  11 Precordialgia [ ] 12. Típica [ ] 13. Angina instável [ ]  14. Náuseas [ ]  15 Fadiga [ ]  16. Cefaléia [ ]  17. Tontura [ ]  18. Síncope [ ]  19. Palpitação [ ] |
| **Hábitos de vida (1-Sim; 2- Não)** |
| 20. Etilismo: [ ] 21. Doses/dia: .................... 22. Tempo de uso:........................ 23. Abstenção:................  24. Tabagismo [ ] 25. Maços/ano: .................... 26. Tempo de uso: ...........................27. Abstenção:...............  28. Atividade física (≥150min/sem de exercício aeróbico) [ ] 29. Tempo:............ |
| **Diagnósticos E PROCEDIMENTOS (1-Sim; 2- Não; 3- Não sabe)** |
| 31. HAS [ ] 32. DM [ ] 33. Dislipidemia [ ]  34. DAC [ ] 35. AVC [ ] 36. ICC [ ]  37. Cardiopatia Congênita [ ] tipo:................................................................... 38. IRC [ ]  39. Doença de Chagas [ ] 40. Febre reumática [ ] 41. FA [ ]  42. Hipertensão pulmonar [ ] 43. Embolia pulmonar [ ] 44. Endocardite [ ]  45. Pericardite [ ] 46. Miocardite [ ] 47. Hemocromatose primária [ ]  48. Hemocromatose secundária [ ] causa:....................................................................................................................  49. Hemoglobinopatia [ ] tipo:......................................................................................................................................  50. Hepatite crônica/cirrose [ ] causa:...........................................................................................................................  51. Outros diagnósticos: ..................................................................................................................................................  52. Revasc. Miocárdica [ ] 53. ATC [ ] 54. Marcapasso [ ]  55. CDI [ ] 56. Plastia/prótese valvar [ ] 57. Flebotomia [ ]  58. Hemotransfusão [ ] quantidade na vida:........................ quantidade nos últimos 3 meses:...........................  59. Outros procedimentos: ...............................................................................................................................................  60. Atendimento de emergência nos últimos 3 meses. **N°...............................................................................................** |
| **HISTÓRIA FAMILIAR (1-Sim; 2- Não; 3- Não sabe)** |
| 61. Morte súbita [ ] 62. Miocardiopatia [ ] 63. DAC familiar precoce [ ] |
| **TRATAMENTO MEDICAMENTOSO (1- Sim; 2- Não; 3- Não sabe)** |
| 64. IECA [ ] Subst.:_________________________________ Dose/dia:_______________  65. BRA [ ] Subst.:_________________________________ Dose/dia:_______________  66. BCC [ ] Subst.:_________________________________ Dose/dia:_______________  67. Beta-bloqueador [ ] Subst.:_________________________________ Dose/dia:_______________  68. Hidroclorotiazida [ ] Subst.:_________________________________ Dose/dia:_______________  69. Furosemida [ ] Subst.:_________________________________ Dose/dia:_______________  70. Espironolactona [ ] Subst.:____________________________________ Dose/dia:_______________  71. Digitálico [ ] Subst.:_________________________________ Dose/dia:_______________  72. Amiodarona [ ] Subst.:_________________________________ Dose/dia:_______________  73. Hidralazina [ ] Subst.:_________________________________ Dose/dia:_______________  74. Nitrato [ ] Subst.:_________________________________ Dose/dia:_______________  75. Antiagregante [ ] Subst.:_________________________________ Dose/dia:_______________  76. Anticoagulante [ ] Subst.:_________________________________ Dose/dia:_______________  77. Estatina [ ] Subst.:_________________________________ Dose/dia:_______________  78. Penicilina Benz. [ ] Subst.:_____________________________________ Dose/mês:_______________  79. Sulfato ferroso [ ] Subst.:_________________________________ Dose/dia:_______________  80. Outros medicamentos: ___________________________________________________ |
| **EXAME FÍSICO** |
| 81. PAS _______mmHg 82. PAD _______mmHg 83. FC_______bpm 84. Peso _________Kg  85. Altura ________m 86. IMC _______Kg/m² 87. Circ.abd. _______cm 88. Quadril _______cm 89. CF ____ |
| **RESSONÂNCIA MAGNÉTICA CARDIOVASCULAR ANTES (1- Sim; 2- Não; 9- Não se aplica)** |
| Data: / / Hora:  142.AE______mm 143.DDVE______mm 144.VDVE______ml 145.DSVE______mm 146.VSVE______ml 147.FEVE______% 148.VDL______mm 149.VDC______mm 150. VDVD______ml 151.VSVD_____ml  152. FEVD______% 153.PP_____ mm 154.Septo____ mm 155.TAPSE______cm 156.Ao asc_______mm  157.AP______mm 158.PSAP______mmHg 159.DC______L/min 160.Massa ventricular_______g  160. Disf. diastólica do VE: [ ] (1-alt. do relax.; 2-pseudonormal; 3-restritivo; 4-não)  161. Alteração segmentar [ ] (Se sim, anotar a região na seção “alteração segmentar”)  162. Valvulopatias: [ ]  163. IM [ ] (1- ausente/mínima; 2- leve; 3- moderada; 4- severa) 164. VR______ml 165. FR______%  166. EM [ ] (1- ausente/mínima; 2- leve; 3- moderada; 4- severa)  167. IAo [ ] (1- ausente/mínima; 2- leve; 3- moderada; 4- severa) 164. VR______ml 165. FR______%  168. EAo [ ] (1- ausente/mínima; 2- leve; 3- moderada; 4- severa)  169. IT [ ] (1- ausente/mínima; 2- leve; 3- moderada; 4- severa)  170. Derrame pericárdico [ ]  171. Pericardite constrictiva [ ]  172. Miocardiopatia não compactada [ ]  173. Outros:________________________________________________________________________________  ___________________________________________________________________________________________  176. Efeito adverso relacionado ao procedimento [ ] (1- não; 2- claustrofobia; 3- outro). Descrição:.......................................................................................................................................................................... |
| **RESSONÂNCIA MAGNÉTICA CARDIOVASCULAR DEPOIS (1- Sim; 2- Não; 9- Não se aplica)** |
| Data: / / Hora:  142.AE______mm 143.DDVE______mm 144.VDVE______ml 145.DSVE______mm 146.VSVE______ml 147.FEVE______% 148.VDL______mm 149.VDC______mm 150. VDVD______ml 151.VSVD_____ml  152. FEVD______% 153.PP_____ mm 154.Septo____ mm 155.TAPSE______cm 156.Ao asc_______mm  157.AP______mm 158.PSAP______mmHg 159.DC______L/min 160.Massa ventricular_______g  160. Disf. diastólica do VE: [ ] (1-alt. do relax.; 2-pseudonormal; 3-restritivo; 4-não)  161. Alteração segmentar [ ] (Se sim, anotar a região na seção “alteração segmentar”)  162. Valvulopatias: [ ]  163. IM [ ] (1- ausente/mínima; 2- leve; 3- moderada; 4- severa) 164. VR______ml 165. FR______%  166. EM [ ] (1- ausente/mínima; 2- leve; 3- moderada; 4- severa)  167. IAo [ ] (1- ausente/mínima; 2- leve; 3- moderada; 4- severa) 164. VR______ml 165. FR______%  168. EAo [ ] (1- ausente/mínima; 2- leve; 3- moderada; 4- severa)  169. IT [ ] (1- ausente/mínima; 2- leve; 3- moderada; 4- severa)  170. Derrame pericárdico [ ]  171. Pericardite constrictiva [ ]  172. Miocardiopatia não compactada [ ]  173. Outros:________________________________________________________________________________  ___________________________________________________________________________________________  176. Efeito adverso relacionado ao procedimento [ ] (1- não; 2- claustrofobia; 3- outro). Descrição:.......................................................................................................................................................................... |
| **ALTERAÇÃO SEGMENTAR**  (Alterações segmentares: 1-normal; 2-hipocinesia; 3-acinesia; 4-discinesia) |
| |  | Alt. seg.  (antes) | Alt. seg.  (depois) | | --- | --- | --- | | Anterior basal | [ ] | [ ] | | Anterolateral basal | [ ] | [ ] | | Inferolateral basal | [ ] | [ ] | | Inferior basal | [ ] | [ ] | | Inferoseptal basal | [ ] | [ ] | | Anteroseptal basal | [ ] | [ ] | | Anterior medial | [ ] | [ ] | | Anterolateral medial | [ ] | [ ] | | Inferolateral medial | [ ] | [ ] | | Inferior medial | [ ] | [ ] | | Inferoseptal medial | [ ] | [ ] | | Anteroseptal medial | [ ] | [ ] | | Anterior apical | [ ] | [ ] | | Lateral apical | [ ] | [ ] | | Inferior apical | [ ] | [ ] | | Septal apical | [ ] | [ ] | | Apical | [ ] | [ ] | |
| **EFEITOS ADVERSOS ASSOCIADOS AO USO DO MEDICAMENTO** |
| Imediatamente após o uso:  177. Cefaléia [ ] 178. *Flushing* [ ] 179. Hipotensão [ ] 180. Turvação visual [ ] 181. Tontura [ ]  182. Epistaxe [ ] 183. Diarréia [ ] 184. Exantema [ ]  185. Outro:________________________________________________________________________________  Uma hora (ou ____ hora) após o uso:  177. Cefaléia [ ] 178. *Flushing* [ ] 179. Hipotensão [ ] 180. Turvação visual [ ] 181. Tontura [ ]  182. Epistaxe [ ] 183. Diarréia [ ] 184. Exantema [ ]  185. Outro:________________________________________________________________________________ |
| **MEDICAMENTO EM USO** |
| 186. Citrato de sildenafil [ ] 187. Placebo [ ] |
| Pesquisador: |
