# Supplementary material for: The Immediate Effect of Sildenafil on Right Ventricular Function in Patients with Heart Failure Measured by Cardiac Magnetic Resonance: A Randomized Control Trial
Source: PLoS One. 2015 Mar 20;10(3):e0119623. doi: 10.1371/journal.pone.0119623 (PMC4368670; doi:10.1371/journal.pone.0119623)
Supplement: S2 Protocol — (DOC) [file pone.0119623.s003.doc]

| 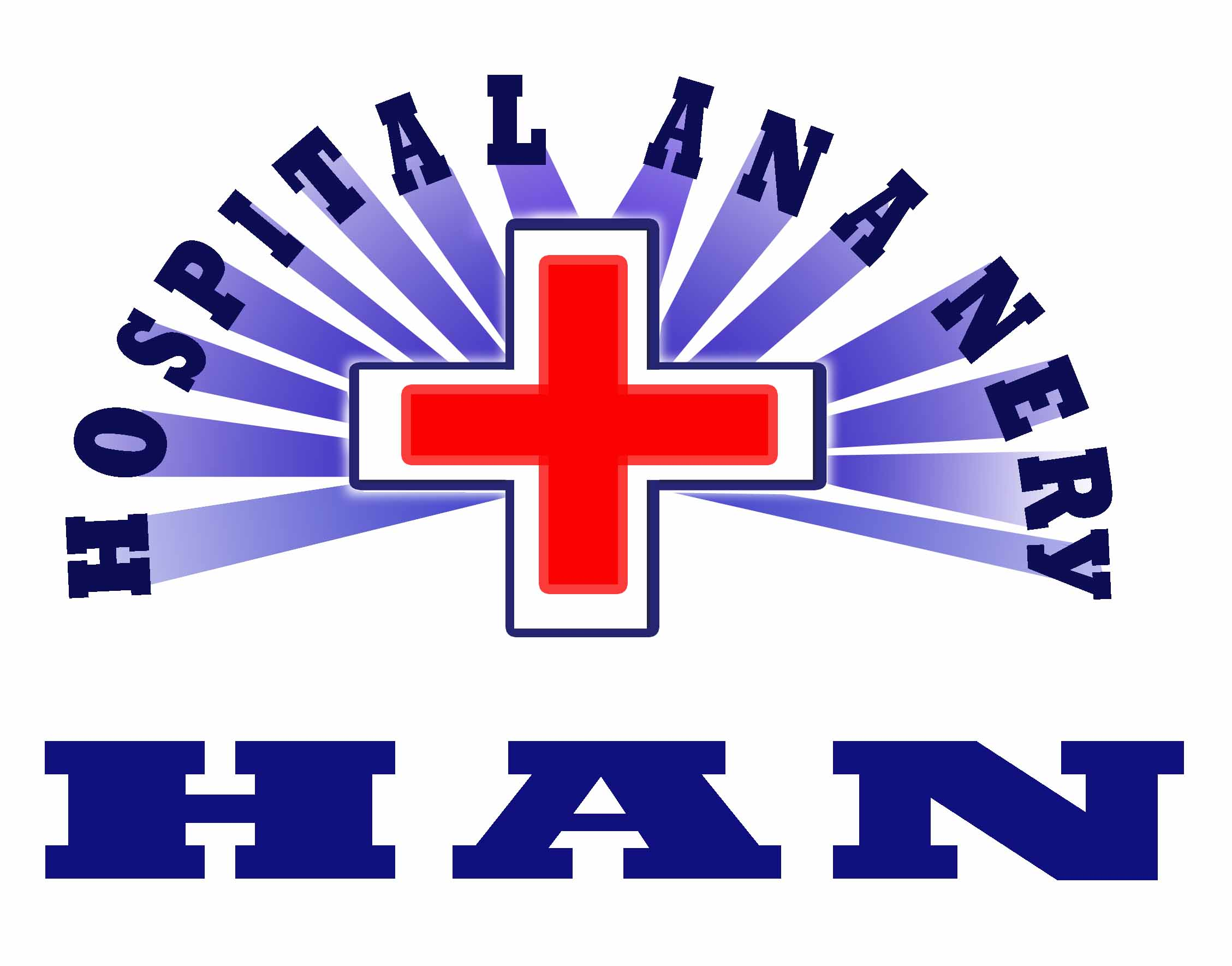 | **HOSPITAL ANA NERI** | **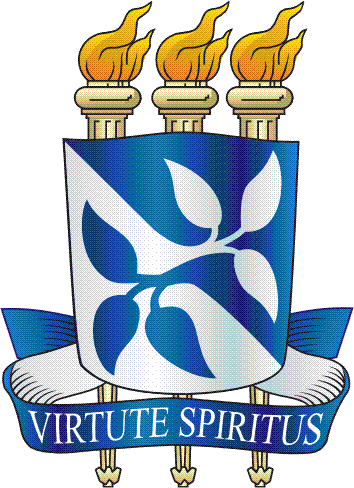** |
| --- | --- | --- |
| HAN |  | **UFBA** |

**Impacto do sildenafil na função ventricular direita de pacientes com insuficiência cardíaca**

PESQUISADOR: André Maurício Souza Fernandes

Salvador - BA

2012

**Título:** **Impacto do sildenafil na função ventricular direita de pacientes com insuficiência cardíaca.**

**Title: Sildenafil influence at right ventricular function in patients with heart failure**

Hospital Ana Neri

Setor de Bioimagem do HAN

- **Pesquisador: ANDRÉ MAURÍCIO SOUZA FERNANDES,** Médico cardiologista. Preceptor da unidade de internação de cardiologia do HAN – UFBA [andremsf@hotmail.com](mailto:andremsf@hotmail.com)
- **Colaboladores:**

**ROQUE ARAS JÚNIOR**, Médico cardiologista. Diretor médico do HAN. Professor adjunto IV do departamento de Medicina da UFBA.

**SIRLENE MENDES BORGES**, Médica radiologista. Coordenadora do setor de bioimagem do HAN.

**ALISSON DE AQUINO FIRMINO**, Biomédico. Funcionário do setor de bioimagem do HAN.

**DAFNE CARVALHO ANDRADE**, Estudante da Faculdade de Medicina da Bahia da UFBA, cursando o quinto ano do curso em 2012.

**IGOR CARMO BORGES**, Estudante da Faculdade de Medicina da Bahia da UFBA, cursando o quinto ano do curso em 2012.

**AGNES CARVALHO ANDRADE**, Estudante da Faculdade de Medicina da Bahia da UFBA, cursando o terceiro ano do curso em 2012.

**NATÁLIA DUARTE BARROSO**, Estudante da Faculdade de Medicina da Bahia da UFBA, cursando o terceiro ano do curso em 2012.

**CARTA DE LIBERAÇÃO**

**SETOR DE BIOIMAGEM DO HOSPITAL ANA NERI**

Ao

Comitê de Ética e Pesquisa (CEP-HAN-UFBA)

Pelo presente, eu, **Sirlene Mendes Borges,** estou de acordo com o trabalho de pesquisa “**Impacto do sildenafil na função ventricular direita de pacientes com insuficiência cardíaca**” a ser realizado no setor de bioimagem do Hospital Ana Neri, sob a coordenação de Dr.André Maurício Souza Fernandes, e aceito participar de todas as fases do processo.

## Salvador, 2012.

.................................................................

**Sirlene Mendes Borges**

**CARTA DE ENCAMINHAMENTO**

Eu, **André Maurício Souza Fernades,** pesquisador do projeto de pesquisa intitulado “**Impacto do sildenafil na função ventricular direita de pacientes com insuficiência cardíaca**” a ser realizado no Hospital Ana Neri, apresento o referido projeto para ser submetido à avaliação desse Comitê, bem como assumo a responsabilidade de acompanhá-lo durante todas as fases de seu desenvolvimento.

## Salvador, de de 2012.

.................................................................

**André Maurício Souza Fernades**

**ÍNDICE**

I. QUESTÃO

II. INTRODUÇÃO

III. OBJETIVOS

IV. MATERIAL E MÉTODOS

V. ASPECTOS ÉTICOS

VI. REFERÊNCIAS BIBLIOGRÁFICAS

VII. EXEQUIBILIDADE

VIII. APLICABILIDADE

IX. IMPACTO

X. ANEXOS

**I. QUESTÃO**

QUAL O IMPACTO DE UMA DOSE ORAL DE 50 MG DE CITRATO DE SILDENAFIL NA FUNÇÃO VENTRICULAR DIREITA DE PACIENTES COM INSUFICIÊNCIA CARDÍACA?

**II. INTRODUÇÃO**

A insuficiência cardíaca congestiva (ICC) é classicamente caracterizada como uma doença progressiva iniciada a partir de dano ao músculo cardíaco e prejuízo à sua força de contração. Contudo, apesar do evento primário estar relacionado especificamente ao coração, essa condição implica em consequências para outros órgãos e sistemas. A hipertensão arterial pulmonar (HAP), neste caso do tipo pós-capilar ou do grupo 2 na classificação de Dana Point, é um exemplo de repercussão extracardíaca da doença. Apesar de ser uma doença pouco frequente na população geral, a HAP apresenta alta prevalência em pacientes com ICC (68-78% em pacientes com disfunção de ventrículo esquerdo [VE] grave), sendo comumente associada à disfunção do ventrículo direito (VD) e considerado um fator de mau prognóstico. Ainda mais, a resistência vascular pulmonar e a performance do VD são considerados importantes determinantes da capacidade cardiorrespiratória de indivíduos com disfunção grave de VE (Ghio, 2001, JACC; Jackle, 1992, JACC; Butler, 1992, JACC; Franciosa, 1985, Am Heart J; Groote, 1999, JACC).

Mesmo que a HAP nesse cenário seja uma consequência das altas pressões de enchimento das câmaras cardíacas esquerdas, o tratamento otimizado para a ICC muitas vezes falha em reverter parâmetros associados à HAP e, atualmente, a terapia da hipertensão pulmonar pós-capilar não tem sido recomendada rotineiramente (Galie, 2009, Eur Heart J). Essa situação expõe a necessidade do desenvolvimento de novas estratégias terapêuticas para a condição.

Via do óxido nítrico (NO)

Nesse contexto, muita atenção tem sido direcionada para intervenções envolvendo a via do NO, um mediador químico importante na regulação do tônus vascular pulmonar e sistêmico. Essa substância é produzida pelo endotélio vascular e age através da sua difusão para a camada muscular da parede vascular, sinalizando o aumento da produção de guanosina monofosfato cíclica (GMPc) dentro das células de músculo liso. O resultado desse aumento de GMPc é o relaxamento das fibras musculares e consequente vasodilatação. Para controlar esse refinado processo de vasodilatação, a disponibilidade intracelular de GMPc é regulada por uma enzima chamada fosfodiesterase 5 (PDE5), que quebra o GMPc em compostos inativos (Levine, 2012, Cardiology). A interação com esse grupo de mediadores tem se dado principalmente pela regulação da biodisponibilidade de NO, através de diferentes abordagens.

Os inibidores da PDE5 são extensamente usados no tratamento da disfunção erétil e da HAP, sendo considerados tratamento de primeira linha para alguns casos deste último. Após numerosos ensaios clínicos e anos de experiência no tratamento dessas duas condições, os inibidores de PDE5, principalmente o sildenafil e o tadalafil, provaram ter um perfil de segurança adequado. Essas substâncias têm mostrado que não aumentam a ocorrência de eventos cardiovasculares (mesmo em pacientes com doença arterial coronariana ou ICC) e que são seguras quando usadas concomitantemente com a maioria das medicações (contraindicação absoluta apenas durante uso de nitratos). É ainda importante que seja destacado que a PDE5 não está confinada à vasculatura peninana e pulmonar. Esta enzima também está presente na vasculatura sistêmica e em células miocárdicas, de modo que os efeitos dessas medicações não são restritos ao pulmão e ao pênis. Inclusive, existem estudos avaliando o uso desta substância no tratamento de condições como infarto do miocárdio, hipertensão arterial sistêmica e fenômeno de Raynaud (Shwartz, 2012, JACC; Archer, 2009, NEJM; Shwartz, 2010, Circulation).

Sildenafil e ICC

Estudos recentes têm mostrado que o uso de uma dose de 50 mg de sildenafil melhora significativamente a pressão arterial pulmonar, a resistência venosa periférica e a pressão capilar pulmonar em pacientes com ICC e HAP (Dumitrescu, 2011, Int J Cardiol; Hirata, 2005, Am J Cardiol; Lewis, 2007, Circulation). Essas alterações hemodinâmicas ainda têm sido associadas a uma melhora da função ventricular direita, da capacidade física e cardiorrespiratória e de outros parâmetros relevantes associados ao prognóstico de pacientes com disfunção sistólica de VD. Além disso, o tratamento crônico com sildenafil tem demonstrado melhora sustentada de parâmetros hemodinâmicos da circulação pulmonar, da capacidade funcional e no remodelamento reverso ventricular direito em pacientes com ICC e HAP (Michelakis, 2003, Circulation).

Um detalhe importante sobre esses estudos é que eles têm avaliado os efeitos cardiovasculares do sildenafil através da utilização de exames ecocardiográficos e/ou invasivos, que não permitem uma avaliação precisa de alguns parâmetros da estrutura e função do coração.

Ressonância magnética cardiovascular

A ressonância magnética tem se tornado um método de imagem bastante utilizado na área da bioimagem, principalmente pela sua elevada acurácia em caracterizar os tecidos, além de dispensar o uso de radiação ionizante, isótopos radioativos ou contrastes iodados. No campo da cardiologia, a alta resolução espacial e temporal da técnica proporcionam avaliações cardiovasculares bastante detalhadas, sendo amplamente utilizadas quando é necessária a realização de medições precisas, especialmente quanto à função, estrutura e componentes tissulares do coração (Guideline RM e TC, 2004; Bradlow, 2012, JCMR). Novas técnicas de obtenção e análise de imagens da RMC ainda têm sido especificamente desenvolvidas para aprimoramento da avaliação da função ventricular direita, permitindo um exame ainda mais meticuloso das câmaras direitas do coração (Kondo, 1992, Radiology; Kind, 2010, JCMR; Sanz, 2009, JACC).

Vale ressaltar que, como todo método, a RMC possui desvantagens. Uma delas é a ocorrência em alguns pacientes de claustrofobia, impossibilitando a permanência no aparelho ou até mesmo a realização do exame. Além disso, existe a necessidade do uso de contraste paramagnético, o gadolínio, em alguns exames, o qual está contra indicado para pacientes com taxa de filtração glomerular menor que 40ml/min. O uso do gadolínio nesses pacientes pode estar associado a fibrose sistêmica nefrogênica, muitas vezes fatal (Kribben, 2009, JACC). Mesmo com essas ressalvas, a RMC é considerada um procedimento bastante seguro.

**III. OBJETIVOS**

**GERAL**

Avaliar o impacto da administração de uma dose oral única de citrato de sildenafil sobre a função ventricular direita de pacientes com insuficiência cardíaca.

**IV. MATERIAL E MÉTODOS**

**1. Local do estudo**

O estudo será realizado no setor de bioimagem do Hospital Ana Neri (HAN), localizado na cidade de Salvador (Bahia). Este serviço é considerado referência no atendimento em doenças cardiovasculares no estado da Bahia.

**2. Métodos**

2.1 Desenho do estudo

Trata-se de um ensaio clínico randomizado duplo cego placebo controlado.

2.2 Cálculo da amostra

O tamanho da amostra calculado para cada grupo foi de 12 participantes, totalizando 24 participantes no estudo. O cálculo amostral foi realizado baseado no estudo de Lewis et al (2007), considerando a diferença a ser detectada na comparação da fração de ejeção do ventrículo direito de 8%, desvio padrão de 4%, nível de significância de 0,1%, poder do teste de 95% e teste de hipótese bicaudado

2.3 Critérios de inclusão:

Pacientes com idade ≥18 anos, com IC crônica, acompanhados no ambulatório de insuficiência cardíaca do Hospital Ana Neri e com disfunção do ventrículo esquerdo (fração de ejeção <35%), disfunção do ventrículo direito e hipertensão arterial pulmonar (PSAP >30 mmHg), avaliados por um ecocardiograma de no mínimo seis meses. Os pacientes envolvidos no estudo também deverão estar com terapia medicamentosa otimizada e mantida na semana precedente à participação no estudo.

2.4 Critérios de exclusão:

Pressão sistólica <90 mmHg ou diastólica <60 mmHg, classe funcional IV (NYHA), presença de incapacidade mental ou psíquica, doença pulmonar obstrutiva crônica, arritmia não controlada, doença valvar mitral moderada a grave, hipersensibilidade a algum componente da droga, uso de nitrato ou nebivolol nas últimas 24 horas à participação no estudo e evento isquêmico coronariano agudo ou exacerbação da insuficiência cardíaca no mês precedente à participação no estudo. Serão também excluídos do estudo pacientes com claustrofobia, implantes metálicos (por exemplo, dispositivo intra-uterino, válvula cardíaca, placa, pino, parafuso, stent, clip de aneurisma cerebral, estilhaço metálico no corpo, piercing, prótese metálica, aparelho ortodôntico) e implantes eletrônicos (marca-passo cardíaco, neuro-estimulador, implante coclear).

2.5 Sequência de atendimento:

Os pacientes responderão a um questionário sobre dados clínicos e sociodemográficos e depois serão submetidos a RMC antes e após 1 hora de uso de 50 mg de citrato de sildenafil. As imagens da RMC serão obtidas utilizando um magneto de corpo inteiro de 1.5T Avanto Siemens. Os indivíduos serão examinados em decúbito dorsal e a recepção do sinal era obtida através de uma bobina de 4 canais. Os seguintes parâmetros serão utilizados: tempo de repetição (TR) = 9ms, tempo de eco (TE) = 4ms, ângulo flip (FA) = 40 graus, espessura de corte = 8mm, número de excitações (NEX) = 2-4, no domínio da view = 380 - 420mm, matriz de 128 x128. Imagens guias em cortes axial, coronal e sagital serão usadas para planejar seqüências multiplanares com precessão livre dos prótons que correspondem a imagens dinamicas do coração (eixo curto, eixo horizontal

longo, vertical longo ). Em nenhum momento do estudo os pacientes serão submetido a radiação ionizante e não haverá uso de qualquer tipo de contraste. Após a aquisição de imagens, serão realizadas as medidas funcionais do ventrículo esquerdo (VE) e do ventrículo direito (VD) (Guideline RM e TC, 2004). Imediatamente e 1 hora após o uso de 50 mg de sildenafil, o paciente será questionado quanto à presença de efeitos adversos ao uso da droga. O médico avaliador da ressonância magnética não terá contato com o paciente e o mesmo será identificado na plataforma do exame por um número anteriormente cadastrado pela equipe de pesquisa. Estando esse, portanto, “cego” ao uso ou não da medicação. O paciente que apresentar intercorrência durante o exame, efeito colateral do uso da medicação ou claustrofobia será atendido pelo médico do setor e excluído da amostra do estudo, já que o paciente deixou de preencher o critério “cego” do estudo.

2.6 Variáveis de interesse:

Dados clínicos e sociodemográficos: idade, sexo, raça, história de sintomas, hábitos de vida, diagnósticos prévios, antecedentes familiares, tratamento medicamentoso, freqüência cardíaca, pressão arterial, peso, altura, medida de cintura abdominal, alergias medicamentosas e presença de reação adversa ao uso de citrato de sildenafil.

Resultado da ressonância magnética antes e após 50mg de sildenafil: fração de ejeção do VE e do VD, volumes sistólico e diastólico do VE e do VD, diâmetros sistólico e diastólico do VE e do VD, análise de contratilidade e torção ventricular, TAPSE (*tricusped annular plane systolic excursion*), massa ventricular esquerda e direita, volume de regurgitação tricúspide e mitral, curvatura septal, pressão no ventrículo direito, débito cardíaco, fluxo pulmonar e distensibilidade da artéria pulmonar.

2.7 Hipóteses:

Hipótese nula: uma dose oral de 50mg de citrato dsildenafil não altera a função ventricular direita de pacientes com insuficiência cardíaca.

Hipótese alternativa: uma dose oral de 50mg de citrato de sildenafil não altera a função ventricular direita de pacientes com insuficiência cardíaca.

**3. Análise estatística**

Serão avaliadas as correlações entre as variáveis clínicas, sociodemográficas e do resultado da ressonância magnética cardiovascular dos pacientes antes e após 50mg de sildenafil. Serão também descritos os dados coletados por meio de médias e desvios-padrões ou números absolutos e números relativos. A análise estatística será desempenhada com auxilio do software SSPS (versão 9.0), com a realização de testes estatísticos como: teste t de Student, Mann Whitney, teste t pareado, teste dos sinais de Wilcoxon, teste qui quadrado, teste exato de Fisher, Kruskall Wallis, one-way ANOVA, teste de correlação de Pearson e regressão linear e logística, simples ou multivariada.

**V. ASPECTOS ÉTICOS**

O trabalho será submetido ao Comitê de Ética em Pesquisa do HAN. Quando na publicação dos resultados, nenhum paciente será identificado e o HAN será citado na publicação, não inferindo qualquer tipo de maleficência ao paciente decorrente aos dados publicados. No final do estudo, será encaminhado ao Comitê de Ética em Pesquisa relatório final e cópia do trabalho que será publicado.

Possíveis efeitos adversos relacionados ao uso de sildenafil incluem cefaléia, *flushing*, hipotensão, turvação visual, tontura, epistaxe, diarréia e exantema. Normalmente, nenhum dos efeitos adversos associados ao uso da droga é grave, não representando perigo para a vida dos participantes do presente estudo. Caso o participante apresente algum sintoma associado ao uso da droga em questão, este será mantido em observação pela equipe do estudo até melhora completa ou estabilização do quadro, sendo encaminhado para o pronto atendimento da instituição para estabilização clínica, caso necessário.

**VI. REFERÊNCIAS BIBLIOGRÁFICAS**

**VII. EXEQUIBILIDADE**

O setor de bioimagem do HAN dispõe de um aparelho de ressonância magnética 1.5T recentemente adquirido, sendo o único aparelho em um hospital exclusivamente público capaz de realizar ressonância magnética cardiovascular no estado da Bahia.

A equipe de pesquisadores dispõe de recurso financeiro pessoal para aquisição das medicações usadas no estudo.

**VIII. APLICABILIDADE**

Ainda não há na literatura estudos mostrando o efeito do citrato de sildenafil na função ventricular direita medida através da RMC.

**IX. IMPACTO**

Imediato. Achados desse estudo podem ter impacto de grande importância no tratamento do pacientes com IC.

**X. APÊNDICES**

Apêndice 01

**CRONOGRAMA**

| **Atividades** | **Mai/12** | **Jun/12** | **Jul/12** | **Jul/12-Dez/12** | **Jan/13** | **Fev/13** | **Mar/13** |
| --- | --- | --- | --- | --- | --- | --- | --- |
| Revisão da literatura | x | x |  |  |  |  |  |
| Construção do projeto e submissao ao CEP | x | x | x |  |  |  |  |
| Coleta de dados |  |  | x | x |  |  |  |
| Análise de dados |  |  |  |  | x | x |  |
| Confecção do manuscrito |  |  |  |  | x | x |  |
| Revisão do manuscrito |  |  |  |  |  | x | x |
| Submissão à publicação |  |  |  |  |  | x | x |

Apêndice 02

**ORÇAMENTO**

Despesas de capital:

1. Aparelho de ressonância magnética de corpo inteiro de 1.5 T Avanto Siemens (disponível no HAN)

2. Notebook LG R480L-3100 c/ Intel® Pentium Dual Core T4300 2.1GHz 3GB 320GB DVD-RW Webcam 1.3MP LED 14" Windows 7 Basic – LG (disponível pelo pesquisador André Maurício Souza Fernandes)

Subtotal: R$ 0

Despesas correntes:

Descrição Qnt Valor Unit Valor Total

Citrato de sildenafil 12 R$ 10,00 R$ 120,00

Placebo 12 R$ 5,00 R$ 60,00

Impressão de material. 50 R$ 0,20 R$ 10,00

Fotocópia 300 R$ 0,10 R$ 30,00

Pasta de arquivos 2 R$ 20,00 R$ 40,00

Subtotal: R$ 260,00

Total: R$ 260,00

Apêndice 03

**TERMO DE CONSENTIMENTO LIVRE E ESCLARECIDO**

Eu,_____________________________________________________________________(nome),______________________(nacionalidade),________(idade), ________________ (estado civil), ___________________(profissão),_______________________________________________________________ (endereço), _______________ (RG), estou sendo convidado a participar de um estudo denominado:

**Impacto do sildenafil na função ventricular direita de pacientes com insuficiência cardíaca**

Fui informado que tenho insuficiência cardíaca, um problema na função de bombear o sangue pelo coração, e que estou sendo convidado a participar de um estudo no qual farei uso de um medicamento que contém citrato de sildenafil ou uma substância sem efeito significativo (plaebo) e também serei submetido a um exame chamado ressonância magnética cardiovascular (um tipo de fotografia especial do coração) antes e uma hora após usar esse medicamento. Este estudo tem como objetivo avaliar o efeito do citrato de sildenafil na função do meu coração.

Fui informado que possíveis efeitos colaterais causados pelo citrato de sildenafil incluem dor de cabeça, vermelhidão na face, pressão baixa, vista embaçada, tontura, sangramento no nariz, diarréia e “empolação” na pele. Informaram também que, normalmente, nenhum dos efeitos colaterais causados pelo medicamento é grave, não representando perigo para minha vida. Fui assegurado que caso eu apresente algum sintoma associado ao uso do medicamento, eu ficarei em observação pela equipe do estudo até melhora completa ou estabilização do quadro, sendo encaminhado para o pronto atendimento do hospital para melhora clínica, caso necessário. Ainda avisaram que caso eu apresente claustrofobia (medo de ficar em lugares fechados) durante o exame de ressonância magnética, o exame será interrompido. O desconforto que poderei sentir durante a entrevista é da possibilidade de compartilhar um pouco das minhas informações pessoais ou confidenciais. Contudo, não precisarei responder qualquer pergunta na entrevista se eu sentir que ela é muito pessoal ou se sentir incômodo ao falar. As informações que estão previstas para serem respondidas no questionário são sobre minha saúde, meus medicamentos e exames que fiz anteriormente.

Estou ciente que minha privacidade será respeitada, ou seja, meu nome ou qualquer outro dado ou elemento que possa, de qualquer forma, me identificar, será mantido em sigilo.

Também fui informado de que posso me recusar a participar do estudo, a qualquer momento, sem precisar justificar, não sofrendo qualquer prejuízo à assistência médica. Os pesquisadores também têm o direito de cancelar minha participação no estudo a qualquer momento.

Foi garantido que terei livre acesso a todas as informações e esclarecimentos adicionais necessários e que o pesquisador se compromete a deixar uma cópia do relatório final da pesquisa nesta instituição para disposição de todos, e fará a divulgação dos resultados obtidos através de eventos e revistas científicas nacionais e internacionais.

Fui informado que todas as despesas com a pesquisa, bem como o ressarcimento de gastos decorrentes dos riscos e complicações causados pelo uso do medicamento e realização da entrevista serão de total responsabilidade do pesquisador. Os dados obtidos serão armazenados por um período de cinco anos. Este termo de consentimento livre e esclarecido será assinado por mim em duas vias, com o compromisso do pesquisador de me proporcionar uma cópia do mesmo para meu controle.

Conforme determina a Resolução 196/96, do Conselho Nacional de Saúde, que trata de aspectos éticos da pesquisa de enfermagem envolvendo seres humanos, o presente estudo requer a participação voluntária dos sujeitos.

Afirmo que a minha participação é voluntária, o meu consentimento para participar da pesquisa foi de livre decisão, não tendo sofrido nenhuma interferência do pesquisador. Estou ciente de que não serei remunerada (o) por este ato, de que poderei solicitar o pesquisador para rever as informações que forneci na entrevista, estando livre para corrigir parte do que foi dito por mim, além de me recusar a continuar participando do estudo a qualquer momento sem causar nenhum prejuízo a minha pessoa e nem a meu futuro profissional.

**Salvador, ____ de _________________ de 2012**

___________________________________ ________________________________

Assinatura do Participante da Pesquisa Assinatura do Pesquisador

Dados do pesquisador

Nome: Dr. André Maurício Fernandes
E-mail: andremsf@hotmail.com

Telefone: 3117-1923/1903

Endereço: Rua Saldanha Marinho, s/n - Caixa d'Água  Salvador - BA, 40320-010

Apêndice 04

**FICHA CLÍNICA**

| **Identificação** |
| --- |
| 1. Data: / /  2. Número do registro [ ] [ ] [ ] [ ] [ ] [ ] Externo [ ]  3. Nome ___________________________________________________________________________  4. Data de Nascimento _______/_______/________  5. Idade _________  6. Gênero [ ] (1-feminino, 2-masculino)  7. Grupo racial [ ] (1-Branco, 2-pardo, 3-negro, 4-outro)  8. Telefone: (____)________________________/(____)_________________________ |
| **Historia de Sintomas (1-Sim; 2- Não) *últimos 3 meses** |
| 9. Dispnéia [ ]  10. Edema de MMII [ ]  11 Precordialgia [ ] 12. Típica [ ] 13. Angina instável [ ]  14. Náuseas [ ]  15 Fadiga [ ]  16. Cefaléia [ ]  17. Tontura [ ]  18. Síncope [ ]  19. Palpitação [ ] |
| **Hábitos de vida (1-Sim; 2- Não)** |
| 20. Etilismo: [ ] 21. Doses/dia: .................... 22. Tempo de uso:........................ 23. Abstenção:................  24. Tabagismo [ ] 25. Maços/ano: .................... 26. Tempo de uso: ...........................27. Abstenção:...............  28. Atividade física (≥150min/sem de exercício aeróbico) [ ] 29. Tempo:............ |
| **Diagnósticos E PROCEDIMENTOS (1-Sim; 2- Não; 3- Não sabe)** |
| 31. HAS [ ] 32. DM [ ] 33. Dislipidemia [ ]  34. DAC [ ] 35. AVC [ ] 36. ICC [ ]  37. Cardiopatia Congênita [ ] tipo:................................................................... 38. IRC [ ]  39. Doença de Chagas [ ] 40. Febre reumática [ ] 41. FA [ ]  42. Hipertensão pulmonar [ ] 43. Embolia pulmonar [ ] 44. Endocardite [ ]  45. Pericardite [ ] 46. Miocardite [ ] 47. Hemocromatose primária [ ]  48. Hemocromatose secundária [ ] causa:....................................................................................................................  49. Hemoglobinopatia [ ] tipo:......................................................................................................................................  50. Hepatite crônica/cirrose [ ] causa:...........................................................................................................................  51. Outros diagnósticos: ..................................................................................................................................................  52. Revasc. Miocárdica [ ] 53. ATC [ ] 54. Marcapasso [ ]  55. CDI [ ] 56. Plastia/prótese valvar [ ] 57. Flebotomia [ ]  58. Hemotransfusão [ ] quantidade na vida:........................ quantidade nos últimos 3 meses:...........................  59. Outros procedimentos: ...............................................................................................................................................  60. Atendimento de emergência nos últimos 3 meses. **N°...............................................................................................** |
| **HISTÓRIA FAMILIAR (1-Sim; 2- Não; 3- Não sabe)** |
| 61. Morte súbita [ ] 62. Miocardiopatia [ ] 63. DAC familiar precoce [ ] |
| **TRATAMENTO MEDICAMENTOSO (1- Sim; 2- Não; 3- Não sabe)** |
| 64. IECA [ ] Subst.:_________________________________ Dose/dia:_______________  65. BRA [ ] Subst.:_________________________________ Dose/dia:_______________  66. BCC [ ] Subst.:_________________________________ Dose/dia:_______________  67. Beta-bloqueador [ ] Subst.:_________________________________ Dose/dia:_______________  68. Hidroclorotiazida [ ] Subst.:_________________________________ Dose/dia:_______________  69. Furosemida [ ] Subst.:_________________________________ Dose/dia:_______________  70. Espironolactona [ ] Subst.:____________________________________ Dose/dia:_______________  71. Digitálico [ ] Subst.:_________________________________ Dose/dia:_______________  72. Amiodarona [ ] Subst.:_________________________________ Dose/dia:_______________  73. Hidralazina [ ] Subst.:_________________________________ Dose/dia:_______________  74. Nitrato [ ] Subst.:_________________________________ Dose/dia:_______________  75. Antiagregante [ ] Subst.:_________________________________ Dose/dia:_______________  76. Anticoagulante [ ] Subst.:_________________________________ Dose/dia:_______________  77. Estatina [ ] Subst.:_________________________________ Dose/dia:_______________  78. Penicilina Benz. [ ] Subst.:_____________________________________ Dose/mês:_______________  79. Sulfato ferroso [ ] Subst.:_________________________________ Dose/dia:_______________  80. Outros medicamentos: ___________________________________________________ |
| **EXAME FÍSICO** |
| 81. PAS _______mmHg 82. PAD _______mmHg 83. FC_______bpm 84. Peso _________Kg  85. Altura ________m 86. IMC _______Kg/m² 87. Circ.abd. _______cm 88. Quadril _______cm 89. CF ____ |
| **RESSONÂNCIA MAGNÉTICA CARDIOVASCULAR ANTES (1- Sim; 2- Não; 9- Não se aplica)** |
| Data: / / Hora:  142.AE______mm 143.DDVE______mm 144.VDVE______ml 145.DSVE______mm 146.VSVE______ml 147.FEVE______% 148.VDL______mm 149.VDC______mm 150. VDVD______ml 151.VSVD_____ml  152. FEVD______% 153.PP_____ mm 154.Septo____ mm 155.TAPSE______cm 156.Ao asc_______mm  157.AP______mm 158.PSAP______mmHg 159.DC______L/min 160.Massa ventricular_______g  160. Disf. diastólica do VE: [ ] (1-alt. do relax.; 2-pseudonormal; 3-restritivo; 4-não)  161. Alteração segmentar [ ] (Se sim, anotar a região na seção “alteração segmentar”)  162. Valvulopatias: [ ]  163. IM [ ] (1- ausente/mínima; 2- leve; 3- moderada; 4- severa) 164. VR______ml 165. FR______%  166. EM [ ] (1- ausente/mínima; 2- leve; 3- moderada; 4- severa)  167. IAo [ ] (1- ausente/mínima; 2- leve; 3- moderada; 4- severa) 164. VR______ml 165. FR______%  168. EAo [ ] (1- ausente/mínima; 2- leve; 3- moderada; 4- severa)  169. IT [ ] (1- ausente/mínima; 2- leve; 3- moderada; 4- severa)  170. Derrame pericárdico [ ]  171. Pericardite constrictiva [ ]  172. Miocardiopatia não compactada [ ]  173. Outros:________________________________________________________________________________  ___________________________________________________________________________________________  176. Efeito adverso relacionado ao procedimento [ ] (1- não; 2- claustrofobia; 3- outro). Descrição:.......................................................................................................................................................................... |
| **RESSONÂNCIA MAGNÉTICA CARDIOVASCULAR DEPOIS (1- Sim; 2- Não; 9- Não se aplica)** |
| Data: / / Hora:  142.AE______mm 143.DDVE______mm 144.VDVE______ml 145.DSVE______mm 146.VSVE______ml 147.FEVE______% 148.VDL______mm 149.VDC______mm 150. VDVD______ml 151.VSVD_____ml  152. FEVD______% 153.PP_____ mm 154.Septo____ mm 155.TAPSE______cm 156.Ao asc_______mm  157.AP______mm 158.PSAP______mmHg 159.DC______L/min 160.Massa ventricular_______g  160. Disf. diastólica do VE: [ ] (1-alt. do relax.; 2-pseudonormal; 3-restritivo; 4-não)  161. Alteração segmentar [ ] (Se sim, anotar a região na seção “alteração segmentar”)  162. Valvulopatias: [ ]  163. IM [ ] (1- ausente/mínima; 2- leve; 3- moderada; 4- severa) 164. VR______ml 165. FR______%  166. EM [ ] (1- ausente/mínima; 2- leve; 3- moderada; 4- severa)  167. IAo [ ] (1- ausente/mínima; 2- leve; 3- moderada; 4- severa) 164. VR______ml 165. FR______%  168. EAo [ ] (1- ausente/mínima; 2- leve; 3- moderada; 4- severa)  169. IT [ ] (1- ausente/mínima; 2- leve; 3- moderada; 4- severa)  170. Derrame pericárdico [ ]  171. Pericardite constrictiva [ ]  172. Miocardiopatia não compactada [ ]  173. Outros:________________________________________________________________________________  ___________________________________________________________________________________________  176. Efeito adverso relacionado ao procedimento [ ] (1- não; 2- claustrofobia; 3- outro). Descrição:.......................................................................................................................................................................... |
| **ALTERAÇÃO SEGMENTAR**  (Alterações segmentares: 1-normal; 2-hipocinesia; 3-acinesia; 4-discinesia) |
| |  | Alt. seg.  (antes) | Alt. seg.  (depois) | | --- | --- | --- | | Anterior basal | [ ] | [ ] | | Anterolateral basal | [ ] | [ ] | | Inferolateral basal | [ ] | [ ] | | Inferior basal | [ ] | [ ] | | Inferoseptal basal | [ ] | [ ] | | Anteroseptal basal | [ ] | [ ] | | Anterior medial | [ ] | [ ] | | Anterolateral medial | [ ] | [ ] | | Inferolateral medial | [ ] | [ ] | | Inferior medial | [ ] | [ ] | | Inferoseptal medial | [ ] | [ ] | | Anteroseptal medial | [ ] | [ ] | | Anterior apical | [ ] | [ ] | | Lateral apical | [ ] | [ ] | | Inferior apical | [ ] | [ ] | | Septal apical | [ ] | [ ] | | Apical | [ ] | [ ] | |
| **EFEITOS ADVERSOS ASSOCIADOS AO USO DO MEDICAMENTO** |
| Imediatamente após o uso:  177. Cefaléia [ ] 178. *Flushing* [ ] 179. Hipotensão [ ] 180. Turvação visual [ ] 181. Tontura [ ]  182. Epistaxe [ ] 183. Diarréia [ ] 184. Exantema [ ]  185. Outro:________________________________________________________________________________  Uma hora (ou ____ hora) após o uso:  177. Cefaléia [ ] 178. *Flushing* [ ] 179. Hipotensão [ ] 180. Turvação visual [ ] 181. Tontura [ ]  182. Epistaxe [ ] 183. Diarréia [ ] 184. Exantema [ ]  185. Outro:________________________________________________________________________________ |
| **MEDICAMENTO EM USO** |
| 186. Citrato de sildenafil [ ] 187. Placebo [ ] |
| Pesquisador: |
