# Supplementary material for: The Immediate Effect of Sildenafil on Right Ventricular Function in Patients with Heart Failure Measured by Cardiac Magnetic Resonance: A Randomized Control Trial
Source: PLoS One. 2015 Mar 20;10(3):e0119623. doi: 10.1371/journal.pone.0119623 (PMC4368670; doi:10.1371/journal.pone.0119623)
Supplement: S1 IRB Approval — (PDF) [file pone.0119623.s005.pdf]

HOSPITAL ANA NERY -
HAN/SESAB

PARECER CONSUBSTANCIADO DO CEP

DADOS DO PROJETO DE PESQUISA

Título da Pesquisa: Impacto do sildenafil na função ventricular direita de pacientes com insuficiência cardíaca

Pesquisador: Andre Maurício Souza Fernandes

Área Temática: Área 3. Fármacos, medicamentos, vacinas e testes diagnósticos novos (fases I, II e III) ou não registrados no país (ainda que fase IV), ou quando a pesquisa for referente a seu uso com modalidades, indicações, doses ou vias de administração diferentes daquelas estabelecidas, incluindo seu emprego em combinações.

Versão: 1

CAAE: 05378712.0.0000.0045

Instituição Proponente: Hospital Ana Nery - HAN/SESAB

DADOS DO PARECER

Número do Parecer: 118.327-2

Data da Relatoria: 27/09/2012

Apresentação do Projeto:

O referido projeto de pesquisa tem como objeto de estudo os pacientes com Insuficiência Cardíaca e que apresentem simultaneamente Hipertensão Pulmonar. Os pacientes do estudo serão submetidos a um medivamento que sabidamente diminui os níveis pressóricos da circulação pulmonar, melhorando assim a função ventricular direita. Este contexto de evolução aguda será avaliado atrvés do método de imagem - Ressonância Nuclear Magnética após a dose de 50mg de Sildenafil.

Objetivo da Pesquisa:

Avaliar a função cardíaca direita de pacientes portadores de Insuficiência Cardíaca que possuam Hipertensão pulmonar através da Ressonância Nuclear Magnética.

Avaliação dos Riscos e Benefícios:

Os riscos de ts pesquisa são mínimos, uma vez que o sildenafil já é um tratamento consolidado na literatura mundial para os pacientes com quadro de Hipertensão Pulmonar. Já em relação a t¿wecnica de imagem, os pacientes que porventura apresentem algum grau de claustrofobia podem evoluir com discreto desconforto.

Comentários e Considerações sobre a Pesquisa:

Este projeto trará dados importantes na análise da função cardíaca direita de pacientes com iC e

que tenham HAP. Não foi encontrado em nenhum passo da realização do projeto de pesquisa nenhum passo que venha a ferir os princípios éticos exigidos pelo CEP.

Considerações sobre os Termos de apresentação obrigatória:

O TCLE se apresenta de forma clara, objetiva e de fácil entendimento para a aplicação durante o trabalho.

Recomendações:

Conclusões ou Pendências e Lista de Inadequações:

O projeto de pesquisa não apresenta nenhuma pendência para sua realização.

Situação do Parecer:

Aprovado

Necessita Apreciação da CONEP:

Não

Considerações Finais a critério do CEP:

SALVADOR, 09 de Outubro de 2012

Assinador por:
Erenaldo de Souza Rodrigues Junior
(Coordenador)

Este parecer reemitido substitui o parecer número 118327-1 gerado na data 10/10/2012 01:08:35.

Endereço: Rua Saldanha Marinho, s/nº
Bairro: Caixa D Água **CEP:** 40.323-010
UF: BA **Município:** SALVADOR
Telefone: (71)3342-2505 **Fax:** (71)3117-1972 **E-mail:** armenio@terra.com.br
